# Identification of epigenetic memory candidates associated with gestational age at birth through analysis of methylome and transcriptional data

**DOI:** 10.1038/s41598-021-83016-3

**Published:** 2021-02-09

**Authors:** Kohei Kashima, Tomoko Kawai, Riki Nishimura, Yuh Shiwa, Kevin Y. Urayama, Hiromi Kamura, Kazue Takeda, Saki Aoto, Atsushi Ito, Keiko Matsubara, Takeshi Nagamatsu, Tomoyuki Fujii, Isaku Omori, Mitsumasa Shimizu, Hironobu Hyodo, Koji Kugu, Kenji Matsumoto, Atsushi Shimizu, Akira Oka, Masashi Mizuguchi, Kazuhiko Nakabayashi, Kenichiro Hata, Naoto Takahashi

**Affiliations:** 1 Department of Pediatrics, The University of Tokyo Hospital, Hongo, Bunkyo-ku, Tokyo, 113-8655 Japan; 2 Department of Maternal-Fetal Biology, National Research Institute for Child Health and Development, Tokyo, Japan; 3 Division of Biomedical Information Analysis, Iwate Tohoku Medical Megabank Organization, Disaster Reconstruction Center, Iwate Medical University, Iwate, Japan; 4 Department of Social Medicine, National Research Institute for Child Health and Development, Tokyo, Japan; 5 Graduate School of Public Health, St. Luke’s International University, Tokyo, Japan; 6 Department of Allergy and Clinical Immunology, National Research Institute for Child Health and Development, Tokyo, Japan; 7 Medical Genome Center, National Research Institute for Child Health and Development, Tokyo, Japan; 8 Department of Molecular Endocrinology, National Research Institute for Child Health and Development, Tokyo, Japan; 9 Department of Obstetrics and Gynecology, The University of Tokyo Hospital, Tokyo, Japan; 10 Department of Neonatology, Tokyo Metropolitan Bokutoh Hospital, Tokyo, Japan; 11 Department of Obstetrics and Gynecology, Tokyo Metropolitan Bokutoh Hospital, Tokyo, Japan; 12 Division of Biomedical Information Analysis, Institute for Biomedical Sciences, Iwate Medical University, Iwate, Japan; 13 Department of Developmental Medical Sciences, The University of Tokyo, Tokyo, Japan

**Keywords:** DNA methylation, Gene expression, Paediatric research

## Abstract

Preterm birth is known to be associated with chronic disease risk in adulthood whereby epigenetic memory may play a mechanistic role in disease susceptibility. Gestational age (GA) is the most important prognostic factor for preterm infants, and numerous DNA methylation alterations associated with GA have been revealed by epigenome-wide association studies. However, in human preterm infants, whether the methylation changes relate to transcription in the fetal state and persist after birth remains to be elucidated. Here, we identified 461 transcripts associated with GA (range 23–41 weeks) and 2093 candidate CpG sites for GA-involved epigenetic memory through analysis of methylome (110 cord blood and 47 postnatal blood) and transcriptional data (55 cord blood). Moreover, we discovered the trends of chromatin state, such as polycomb-binding, among these candidate sites. Fifty-four memory candidate sites showed correlation between methylation and transcription, and the representative corresponding gene was *UCN*, which encodes urocortin.

## Introduction

Gestational age (GA) and birth weight, particularly low birth weight, are the most important predictors associated with short- and long-term neonatal adverse outcomes. Low birth weight infants can be classified as either preterm infants or small-for-gestational-age (SGA) infants. Preterm infants are defined as those born before 37 weeks of gestation. They are forced to survive *ex utero* midst their fetal development, receiving no direct nutrient and oxygen supply from their mothers, earlier than term infants. In contrast, SGA infants tend to be exposed to hypoxia and malnutrition in utero. Despite differences in etiology and exposure between these two conditions of newborns, both involve disturbed oxygen and nutrition during the perinatal period. To date, there is growing epidemiological evidence that these newborns may be at higher risk of chronic diseases in later life, including coronary heart disease, type 2 diabetes, metabolic syndromes, and neurobehavioral problems^1–3^; higher risk of mortality from coronary heart disease has also been reported^2^. In addition, preterm and/or low birth weight infants are prone to metabolic shift including BMI gain^4^, lower insulin sensitivity^5^, and higher blood pressure^6^compared to normal birth weight infants, even in later childhood. Another line of evidence from the Dutch famine birth cohort studies showed that maternal undernutrition during pregnancy caused high morbidity of offspring in adulthood^7–9^. These findings support the Developmental Origins of Health and Disease (DOHaD) hypothesis^10,11^ which describes that the adaptation for surviving harsh environment in early life may influence the susceptibility to chronic diseases in adulthood^10,11^. In other words, restriction of developmental plasticity may contribute to these personal traits^12^. Considering that epigenetic mechanisms play important roles in tissue differentiation and developmental plasticity^11,12^, epigenetic memory formed in early development may therefore act upon pathways to chronic diseases in later life. However, this hypothesis has not been well-elucidated in humans.

Owing to advances in microarray technology, epigenome-wide association studies (EWAS) are now commonly conducted. In the perinatal field, previous studies have investigated methylation alterations related to GA^13–17^, birth weight^15,18^, and birth weight standard deviation (SD) scores for GA^19^ by using cord blood samples. However, these studies have not examined whether DNA methylation changes relate to RNA expression levels, and those that were able to examine postnatal blood methylation showed inconsistent results. Two previous studies reported that certain methylation changes identified at birth among preterm or low birth weight infants were no longer observed by adulthood^15,20^; however, the results of postnatal methylation persistence were inconsistent^15,20^. Cruickschank et al. suggested that some methylation alterations among preterm infants may persist into adulthood^20^. In contrast, no persistence was observed from the age of 7 years in the report by Simkin et al. in the ARIES cohort study^15^.

The objectives of the current study were to investigate epigenetic alterations associated with preterm birth and SGA through DNA methylation and gene expression microarrays, as well as, to identify epigenetic at-birth changes which may persist as personal traits after birth. The evaluation of DNA methylation, gene expression, and their relationship was performed using both genomic DNA and total RNA samples purified simultaneously. This is the first EWAS study targeting Japanese preterm and/or SGA infants.

## Results

We generated normalized DNA methylation data from 110 cord blood samples and 47 postnatal peripheral blood samples, as well as, normalized gene expression data from 55 cord blood samples as described in Methods, Supplementary Methods, and Supplementary Figures 1–4. The results are described in the order shown in ‘overall analysis framework’ (Supplementary Figure 5). Among the 110 mother-infant pairs included in these analyses, mean GA was 34.0 weeks and mean birth weight SD score was − 0.6 (Table 1, Supplementary Table 1); 34.5% (n = 38) were small-for-GA (SGA; defined as birth weight < 10th percentile, equivalent to − 1.28 SD) and 10.9% (n = 12) were large-for-GA (LGA; defined as birth weight > 90th percentile, equivalent to 1.28 SD). Approximately 81% (n = 89) of total deliveries were by cesarean section. Only 2 mothers (1.8%) smoked during pregnancy, and 7 mothers (6.4%) had smoked before pregnancy. Further, 3.6% of mothers (n = 4) experienced gestational diabetes mellitus, 22.7% (n = 25) had chorioamnionitis, 10% (n = 11) had idiopathic premature rupture of the membrane without inflammation (hereinafter referred to as iPROM), and 18.2% (n = 20) experienced preeclampsia.

**Figure 1:**
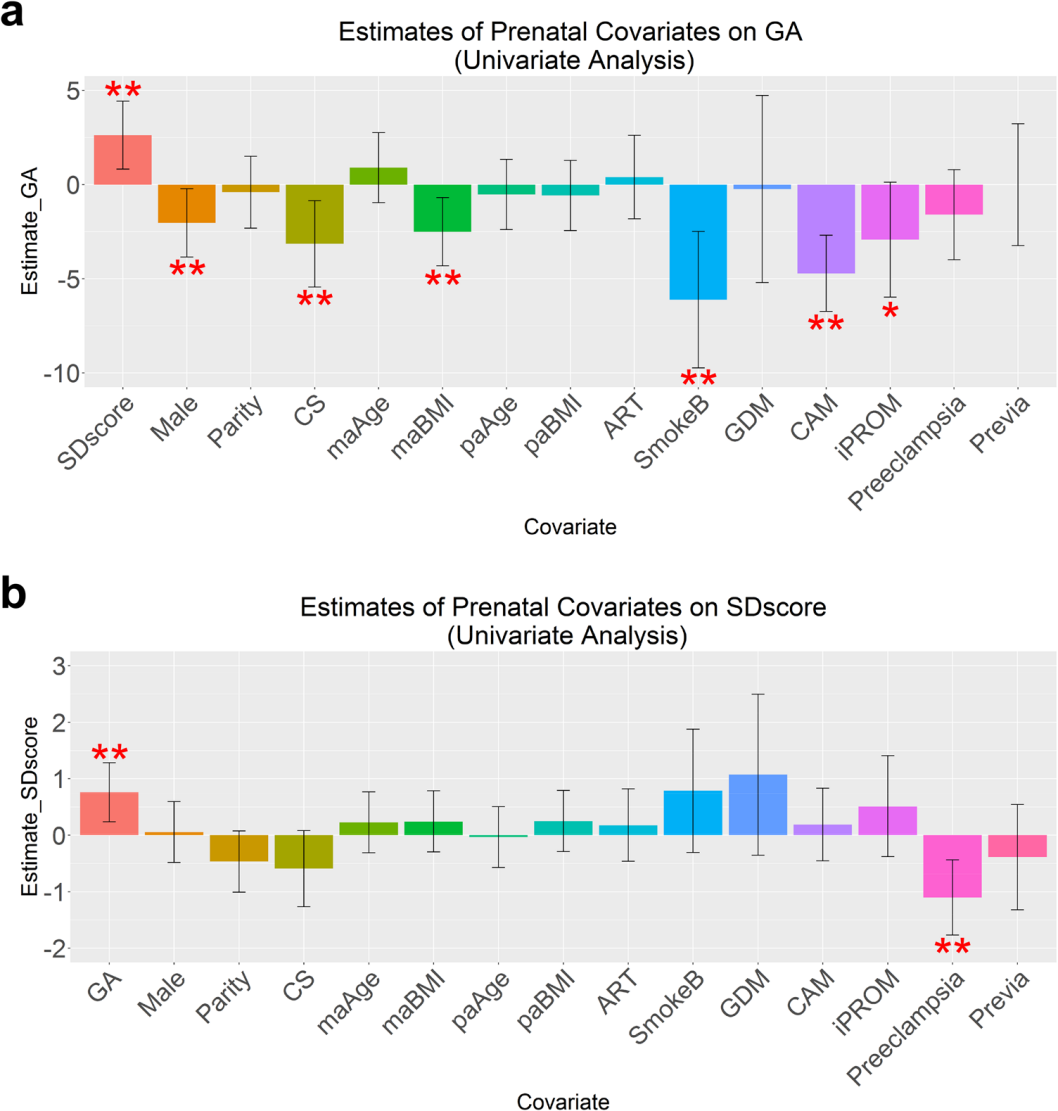
Association of prenatal covariates with gestational age and/or birth weight SD scores (n = 110, cord blood samples). (**a**) Association of prenatal covariates with gestational age (GA). Red asterisks with each covariate represent *p*-values for association between predictor (GA or birth weight SD score) and prenatal covariate (double red asterisks = *p* value < 0.05 (univariate linear regression analysis); single red asterisk = *p* value of 0.060 (suggestive)). Regression coefficients (Estimates) and *p* values are reported as a week’s change in GA for two standard deviation increases in continuous prenatal variables, or for comparing the two categories of binary prenatal variables. Error bars indicate 95% confidence interval of effect size. (**b**) Associations of prenatal covariates with birth weight SD scores. Estimates are reported as changes in birth weight SD scores for two standard deviation increases in continuous prenatal variables, or for comparing the two categories of binary prenatal variables. Abbreviations & which covariate is continuous or binary: [Continuous covariates] *GA*: gestational age, *SD score*: birth weight SD score, *maAge*: maternal age, *maBMI*: maternal pre-pregnancy BMI, *paAge*: paternal age, *paBMI*: paternal BMI [Binary covariates] *Male*, *Parity* (> 0 or 0), *CS*: cesarean section, *ART*: assisted reproductive technology, *SmokeB*: maternal smoking before pregnancy, *GDM*: gestational diabetes mellitus, *CAM*: chorioamnionitis, *iPROM*: idiopathic premature rupture of the membrane, *Preeclampsia*, *Previa*: placenta previa.

**Table 1:** Pregnancy- and delivery-related characteristics of 110 mother-infant pairs.

Prenatal variable	Mean (SD)	Median	N (%)
Sex (Male)			53 (48.2)
Maternal age	33.8 (4.7)	34	
< 25 years			1 (0.9)
25 ~ 30 years			20 (18.2)
30 ~ 35 years			41 (37.3)
35 ~ 40 years			34 (30.9)
> 40 years			14 (12.7)
Maternal pre-pregnancy BMI	21.1 (3.5)	20.3	
< 18.5 kg/m^2^			23 (20.9)
18.5 ~ 25 kg/m^2^			77 (70.0)
25 ~ 30 kg/m^2^			4 (3.6)
> 30 kg/m^2^			6 (5.5)
Parity (> 0)			43 (39.1)
Smoking during pregnancy (Yes)			2 (1.8)
Smoking before pregnancy (Yes)			7 (6.4)
Assisted reproductive technology (ART: Yes)			25 (22.7)
Gestational diabetes mellitus (Yes)			4 (3.6)
Preeclampsia (Yes)			20 (18.2)
Placenta previa (Yes)			10 (9.1)
Chorioamnionitis (CAM: Yes)			25 (22.7)
Idiopathic premature rupture of the membrane (iPROM: Yes)			11 (10.0)
Delivery mode (cesarean section)			89 (80.9)
Gestational age at birth	34.0 (4.9)	35	
< 28 weeks			17 (15.5)
28 ~ 32 weeks			17 (15.5)
32 ~ 37 weeks			31 (28.2)
> 37 weeks			45 (40.9)
Birth weight SD score	− 0.6 (1.4)	− 0.5	
< − 2.5			16 (14.5)
− 2.5 to − 1.28			22 (20.0)
− 1.28 to 1.28			60 (54.5)
1.28 ~ 2.5			12 (10.9)

*Since the table containing all the values exceeds one page, other descriptive characteristics that cannot be written in Table are shown in Supplementary Table 1.

### Covariates associated with GA and/or birth weight SD scores

We evaluated the association between pregnancy- and delivery-related variables, infant sex, and GA at birth and/or birth weight SD scores for GA (hereinafter referred to as SD scores). Higher SD scores were associated with older GA (Fig. 1, Supplementary Tables 2, 3; *p* < 0.05). Male infants, cesarean section, higher maternal pre-pregnancy BMI, maternal smoking before pregnancy, and chorioamnionitis were all associated with earlier GA (Fig. 1a; *p* < 0.05). Moreover, there was a suggestive association between iPROM and earlier GA (Fig. 1a; *p* = 0.060), and preeclampsia was associated with lower SD scores (Fig. 1b; *p* < 0.05). In multivariate analysis that considered these variables, the direction of effect remained similar, and the association with iPROM and preeclampsia became stronger (Supplementary Table 4, 5).

### Epigenome-wide association study on GA and/or birth weight SD scores using cord blood samples and pathway analysis

**Figure 2:**
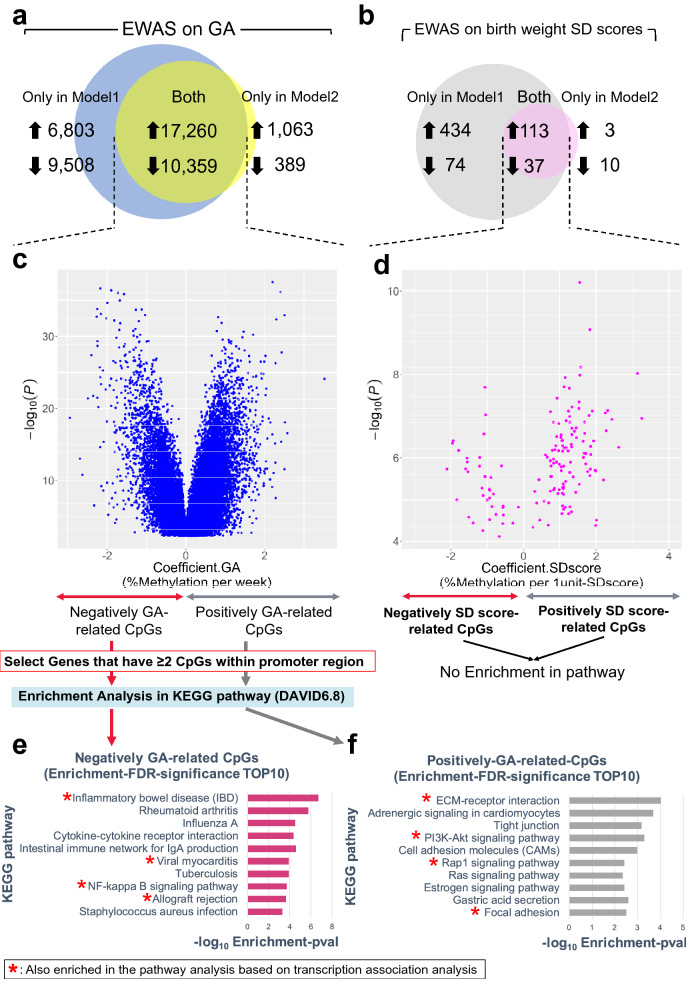
Epigenome-wide association study (EWAS) of gestational age and/or birth weight SD scores (n = 110, cord blood samples). (**a**) Blue circle reflects CpGs associated with gestational age (GA) in “Model 1” linear regression analysis and yellow circle reflects CpGs in “Model 2”. The intersection of blue and yellow circles means finally decided CpGs associated with GA. Upward (or downward) arrows mean the number of CpGs whose methylation increases (or decreases) when predictor values increase. (**b**) Gray circle reflects CpGs associated with birth weight SD score in Model 1 linear regression analysis and pink circle reflects CpGs in Model 2. The intersection of gray and pink circles means finally decided CpGs associated with birth weight SD score. (**c**) Volcano plot indicating regression coefficients (x-axis) versus *p*-values (-log_10_ scale) of CpGs associated with GA. All values were generated in Model 1 analysis. (**d**) Volcano plot indicating regression coefficients (x-axis) versus *p* values (-log_10_ scale) of CpGs associated with birth weight SD scores. All values were generated in Model 1 analysis. (**e**) Top 10 KEGG pathway categories enriched in the gene set of negatively GA-related CpGs by using DAVID 6.8. We selected genes in which the promoter region had at least 2 CpGs associated with GA. Single red asterisk denotes a pathway category which was also enriched in GA-related transcripts. x-axis of the barplots means –log_10_ enrichment *p*-value. (**f**) Top 10 KEGG pathway categories enriched in the gene set of positively GA-related CpGs by using DAVID 6.8. *Model 1) *objective variable*: %methylation, *predictors*: GA, SD-score, *adjusted for*: infant sex, batch, cell proportion. **Model 2) *objective variable*: %methylation, *predictors*: GA, SD-score, *adjusted for*: infant sex, batch, cell proportion, chorioamnionitis, idiopathic premature rupture of the membrane, preeclampsia, maternal smoking before pregnancy, maternal pre-pregnancy BMI, cesarean section. ***GA: gestational age, *SD-score*: birth weight SD score, *EWAS*: epigenome-wide association study.

The EWAS of GA and SD scores using the cord blood samples utilized two linear regression models that differed in the extent of covariate adjustment. Using the false discovery rate (FDR) correction^21^ for multiple testing (q < 0.05, 410,735 tests), based on “Model 1” we identified 43,930 CpG sites associated with GA and 658 CpGs associated with SD score (Fig. 2a,b; Supplementary Fig. 6a, 7a). Based on “Model 2” that adjusted for additional covariates, we identified 29,071 sites associated with GA and 163 sites associated with SD score (Fig. 2a,b; Supplementary Fig. 6b, 7b). We considered candidate CpGs as those associated with GA or SD scores in both models in the same direction, resulting in the identification of 27,619 GA-related CpGs and 150 SD score-related CpGs (Supplementary Tables 6 and 7). We pursued a sensitivity analysis approach used similarly in previous studies to assess whether associations were captured sufficiently from ”Model 2″ which contained six additional prenatal covariates^22^. Regression analysis were pursued adjusting for “Model 1″ covariates in addition to each of these six covariates, in turn, and the number of associated CpGs were observed. Results of all sensitivity analyses (FDR < 0.05) showed overlap of 26,202 GA-related CpGs (95%) and 141 SD score-related CpGs (94%), all of which were included in the larger set of 27,619 GA-related and 150 SD score-related CpGs (Supplementary Table 8, 9). Additionally, we categorized both groups of CpGs based on directionality of the regression coefficients. GA-related CpGs consisted of 17,260 positively related sites and 10,359 negatively related sites (Fig. 2a,c; Supplementary Table 6). SD score-related CpGs consisted of 113 positively related sites and 37 negatively related sites (Fig. 2b,d; Supplementary Table 7). GA-related CpGs were more likely to be located in CpG island shores (*p* < 2.2e−16), the distribution of which was 1.4-fold more than that of all CpGs contained in the HumanMethylation450 BeadChip (hereinafter referred to as 450 k array) (Supplementary Fig. 8). The distribution of SD-score-related CpGs at open sea (*p* = 6.9e−08) was 1.5-fold more than that of all CpGs contained in 450 k array.

For KEGG pathway enrichment analysis, we selected genes in which the promoter region had at least 2 CpGs associated with GA or SD scores. The DAVID bioinformatics resources^23^ found no FDR-significant associations with genes containing SD score-related CpGs (Fig. 2d). Regarding GA-related CpGs, 9 pathway categories were significantly enriched in the analysis for positively GA-related CpGs, and 27 pathway categories were significantly enriched in the analysis for negatively GA-related CpGs after filtering for enrichment-FDR ≤ 0.1 (Supplementary Table 10). The terms indicating inflammation (for example, “inflammatory bowel disease”), “cytokine-cytokine receptor interaction”, and “NF-kappa B signaling pathway” were enriched in the analysis for negatively GA-related CpGs, whereas “ECM-receptor interaction” and “PI3K-Akt signaling pathway” were enriched in the analysis for positively GA-related CpGs (Fig. 2e,f).

### Association analysis of transcription and GA and/or birth weight SD scores and pathway analysis

**Figure 3:**
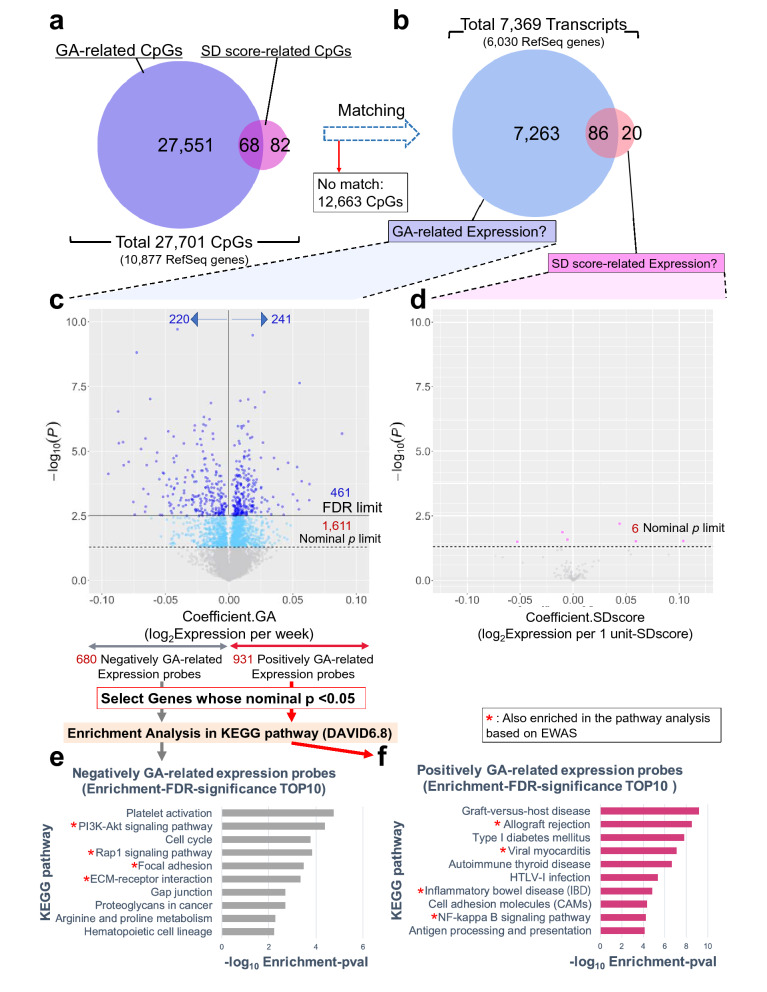
Association analysis of transcription and gestational age and/or birth weight SD score (n = 55, cord blood samples). (**a)** Blue circle reflects CpGs associated with gestational age in cord blood EWAS and pink circle reflects CpGs associated with birth weight SD scores. (**b)** 19,640 among 27,701 CpGs detected in cord blood EWAS were matched to 9,691 gene expression microarray probes. Light blue circle reflects gene expression probes matched to GA-related CpGs, and pink circle reflects probes matched to SD score-related CpGs. (**c)** Volcano plot indicating regression coefficients (x-axis) versus *p*-values (-log_10_ scale) of gene expression probes associated with GA. All values were generated in the multivariate linear regression analysis*. Deep blue dots mean FDR-significant. Light blue dots mean nominal *p* < 0.05 and FDR ≥ 0.05. Grey dots mean nominal *p* ≥ 0.05. (**d)** Volcano plot indicating regression coefficients (x-axis) versus *p*-values (-log_10_ scale) of gene expression probes associated with birth weight SD scores. All values were generated in Model1 analysis. Pink dots mean nominal *p* < 0.05 and FDR ≥ 0.05. Grey dots mean nominal *p* ≥ 0.05. (**e)** Top 10 KEGG pathway categories enriched in negatively GA-related expression probes by using DAVID 6.8. Here, threshold of selecting expression probes is nominal *p* < 0.05. Single red asterisk denotes a pathway category which was also enriched in GA-related CpGs. x-axis of the barplots means –log_10_ enrichment *p*-value. (**f)** Top 10 KEGG pathway categories enriched in positively GA-related expression probes by using DAVID 6.8. *Analysis model) *objective variable*: log_2_-transformed expression of each gene expression probes, *predictors*: GA, SD score, *adjusted for*: infant sex, batch, cell proportion. **GA: gestational age, *SD score*: birth weight SD score, *EWAS*: epigenome-wide association study.

Among the 27,701 CpGs associated with GA and/or birth weight SD scores in the cord blood EWAS, we matched 15,038 CpGs to 7,369 QC-filtered gene expression probes within a region of 250 kb upstream or downstream of CpGs (Fig. 3a,b). Association analysis was performed on these 7,369 transcripts which resulted in the identification of 461 FDR-significant GA-related transcripts (1,611 nominally significant transcripts; nominal *p*-value < 0.05). Among these GA-related transcripts, 220 were negatively related (680 at a nominal *p* < 0.05) and 241 were positively related (931 at a nominal *p* < 0.05) (Fig. 3c; Supplementary Table 11). In contrast, there were no FDR-significant transcripts associated with birth weight SD score, but six were nominally significant transcripts (Fig. 3d; Supplementary Table 12).

When we conducted pathway enrichment analysis for FDR-significant GA-related transcripts, five pathway categories were significant all of which were confined to only the negatively GA-related transcripts (enrichment-FDR ≤ 0.1) (Supplementary Table 13). Enrichment analysis applied to the nominally significant GA-related transcripts resulted in 8 significantly enriched pathway categories among the negatively GA-related expression genes, and 20 significantly enriched pathway categories among the positively GA-related sites (Supplementary Table 14). Some pathway categories were simultaneously ranked within the list of top 10 enriched pathways appropriately in opposite directions in the GA-CpG methylation analysis and GA-expression analysis. The following pathway categories were ranked in both the top 10 lists of negatively GA-related CpGs and positively GA-related transcripts: inflammatory bowel disease, viral myocarditis, NF-kappa B signaling pathway, and allograft rejection. In contrast, the following pathway categories ranked in both top 10 lists of positively GA-related CpGs and negatively GA-related transcripts: ECM-receptor interaction, PI3K-Akt signaling pathway, Rap1 signaling pathway, and focal adhesion (Figs. 2e,f, 3e,f; Supplementary Table 14).

### Confirmation of direct association between DNA methylation and gene expression with methylation expression analysis

**Figure 4:**
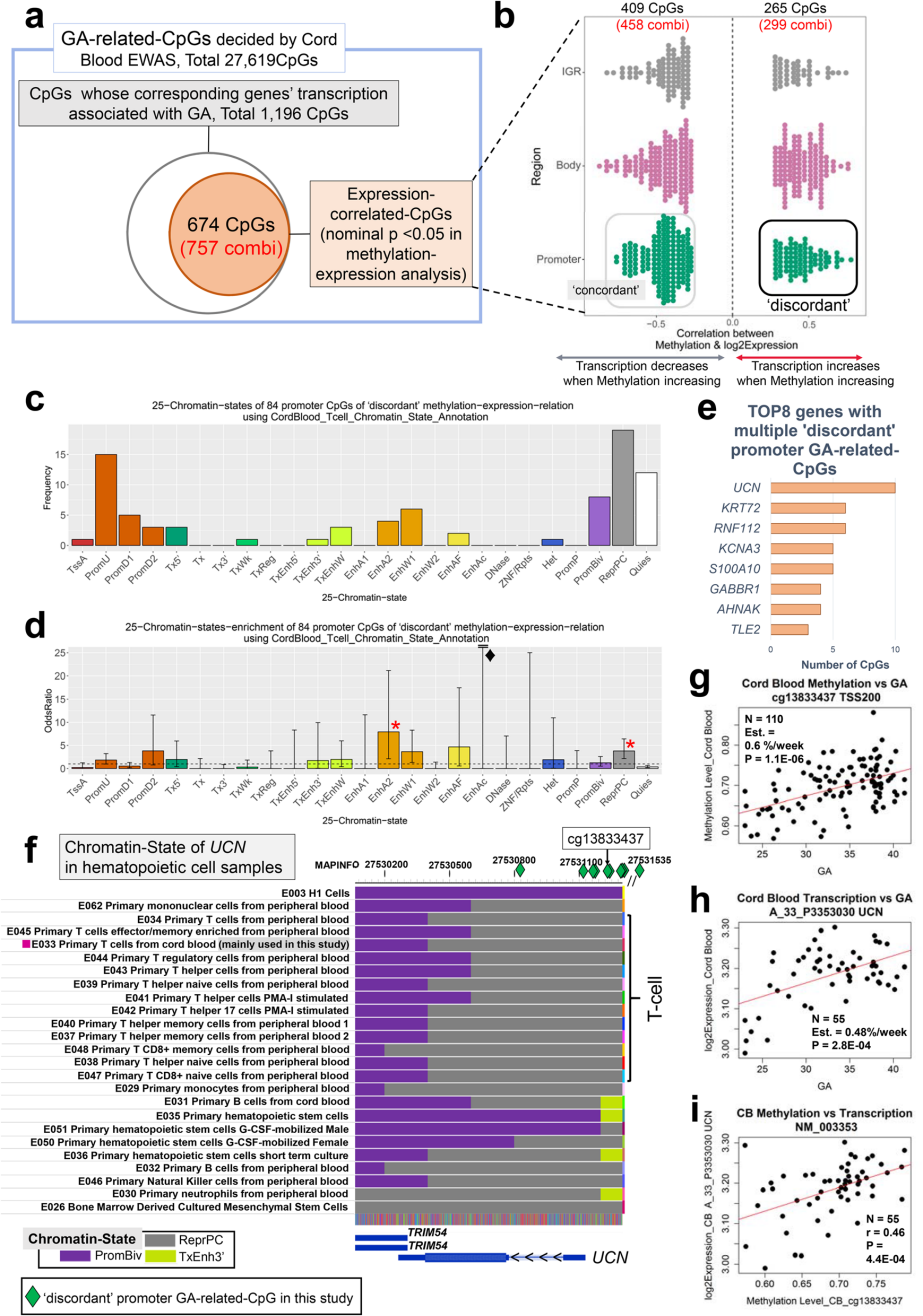
CpG sites whose methylation correlated with corresponding transcription among gestational age-related CpGs. (**a)** Larger circle with gray line reflects 1,196 CpGs whose corresponding genes’ transcription associated with gestational age. Smaller orange circle means 674 CpG sites whose methylation correlated with corresponding transcription log_2_-transformed among the 1,196 CpGs (nominal *p*-value < 0.05; n = 55). ‘Combi’ means the combinations of the same CpGs and those corresponding transcripts. (**b**) Dot plot indicating the distribution of correlation coefficients between methylation and log_2_-transformed gene expression in each of the 3 large regions for the 674 expression-correlated GA-related CpGs. Green dots mean CpGs within “Promoter Region” including TSS1500, TSS200, 5′UTR, 1st Exon. Pink dots mean CpGs within “Gene Body” region including Body, 3′UTR. Black dots mean IGR, *i.e.*, the intergenic region. Regarding “Promoter Region”, we defined negative correlation as ‘concordant’, while positive correlation as ‘discordant’. (**c**) Distribution of 25 chromatin states for 84 promoter GA-related CpGs of ‘discordant’ methylation expression-relation using cord blood T cell-based annotation of ChromHMM. (**d**) Enrichment of 25 chromatin states for 84 promoter GA-related CpGs of ‘discordant’ methylation expression relation. Error bars indicate 95% CI (confidence interval). Single red asterisk denotes the enriched chromatin state which was significant at Bonferroni-criteria (0.05/25) and of odds ratio ≥ 1 (black dashed line). Filled black diamnod indicate that the upper limit of 95% CI is too high to show in error bars because this value is a prominent outlier, more than 3 times of the 2nd highest value and there is no statistical significance. (**e)**. Top 8 genes that have multiple GA-related CpGs of ‘discordant’ methylation expression relation. **f**. Chromatin state of *UCN* in hematopoietic cell samples. Filled green diamnod means a ‘discordant’ promoter GA-related CpG, and the representative is cg13833437. (**g)**. A scatterplot of GA (x-axis) versus cord blood methylation at cg13833437. (n = 110) (**h**) A scatterplot of GA (x-axis) versus log_2_-transformed gene expression of A_33_P3353030, *UCN* transcript (n = 55). (**i**) A scatterplot of methylation of cg13833437 (x-axis) versus log_2_-transformed gene expression of A_33_P3353030 (n = 55). *GA: gestational age. **Chromatin state abbreviations are defined in ChromHMM. Following Abbreviations are defined in ChromHMM; TssA: Active TSS, PromU: Promoter upstream TSS, PromD1: Promoter downstream with DNase, PromD2: Promoter downstream TSS, Tx5′: Transcription 5′, Tx: Transcription, Tx3′: Transcription 3′, TxWk: Weak Transcription, TxReg: Transcription Regulatory, TxEnh5′: Transcription 5′ Enhancer, TxEnh3′: Transcription 3′ Enhancer, TxEnhW: Transcription Weak Enhancer, EnhA1: Active Enhancer 1, EnhA2: Active Enhancer 2, EnhAF: Active Enhancer Flank, EnhW1: Weak Enhancer 1, EnhW2: Weak Enhancer 2, EnhAc: Enhancer Acetylation Only, DNase: DNase only, ZNF/Rpts: ZNF genes & repeats, Het: Heterochromatin, PromP: Poised Promoter, PromBiv: Bivalent Promoter, ReprPC: Repressed Polycomb, Qies: Quiescent/Low.

Based on the results of cord blood EWAS and association analysis of transcription, we generated 1,355 CpG-transcript combinations connected with 461 GA-related expression probes and 1,196 GA-related CpGs within 414 RefSeq genes. From the cord blood samples, 55 were available for investigating direct methylation-expression relationships (Supplementary Fig. 9a). Within the 1,355 CpG-transcript combinations, significant associations between CpG methylation and gene expression were confirmed in 757 combinations (nominal *p* < 0.05) (Supplementary Fig. 9c, Supplementary Table 15). This corresponded to 674 GA-related CpGs showing significant methylation expression correlations to 281 RefSeq genes (Fig. 4a). Among these 674 CpG sites, 409 CpGs (458 combinations) showed negative correlation between % methylation and log_2_ expression values, while 265 CpGs (299 combinations) showed a positive correlation (Fig. 4b). Within the 674 CpG sites, 84 in the promoter regions (including TSS200, TSS1500, 5′UTR, 1st Exon) showed positive correlation between % methylation and log_2_ expression, thus exhibiting a ‘discordant’ relation^24^ where transcription increased when methylation increased (Fig. 4b, green dots). In comparison, ‘concordant’ relation was confirmed in 165 promoter CpGs, where transcription decreased when methylation increased. Within the ‘discordant’ promoter CpGs, “Repressed Polycomb” occupied the highest proportion (22.6%) among the 25 chromatin states of cord blood T cell-based annotation imputed by ChromHMM^25^, with an enrichment odds ratio of 3.8 when compared to the reference proportion based on all CpGs contained in 450 k array (Fig. 4c,d; Supplementary Table 16). We listed the genes in which methylation levels were related to GA at multiple CpGs within the same genes from 84 discordant promoter CpGs (Fig. 4e). *UCN* was the top-ranked gene based on the largest number of CpGs included in the 84 discordant promoter CpGs, and whose promoter was occupied by “Repressed Polycomb” in most types of blood cells except for hematopoietic stem cells (Fig. 4f., E035, E051). Promoters in hematopoietic stem cells were occupied by bivalent promoter states which was in common with H1 embryonic stem cells (Fig. 4f., E003). Indeed, both the methylation in the promoter CpG of *UCN* (cg13833437) and the expression of *UCN* were significantly correlated to GA (Fig. 4g,h)*,* in addition to positive correlation between methylation and expression levels (Fig. 4i). Within the 165 ‘concordant’ promoter CpGs, “Promoter upstream TSS” occupied the highest proportion (22.4%) among the 25 chromatin states of cord blood T cell-based annotation imputed by ChromHMM, with an enrichment odds ratio of 2.4 when compared to the reference proportion based on all CpGs contained in the 450 k array (Supplementary Table 17). *CARD11* was the top-ranked gene based on the largest number of CpGs included in the 165 ‘concordant’ promoter CpGs (Supplementary Fig. 10), and *CARD11* promoter was occupied by “Promoter upstream TSS”.

### Candidate CpGs for GA-involved epigenetic memory

To evaluate whether GA at birth was still associated with DNA methylation in postnatal peripheral blood cells, we conducted EWAS using postnatal blood DNA methylation values. Postnatal blood samples for analysis were collected around their expected due dates from 47 babies whose cord blood samples were utilized for DNA methylation analysis. The GA EWAS was repeated among these 47 cord blood samples, and association with GA at birth was also examined in relation to DNA methylation in their postnatal samples. We identified 8,484 and 0 FDR-significant CpGs associated with GA in cord blood and postnatal peripheral blood, respectively. The volcano plot showing the regression coefficients of the DNA methylation and GA association was V-shaped for the 47 cord blood samples analyzed (Supplementary Fig. 11a), similar to the analysis using 110 samples (Fig. 2c). In contrast, the volcano plot based on the postnatal peripheral blood samples indicated no evidence of an association (Supplementary Fig. 11b).

**Figure 5:**
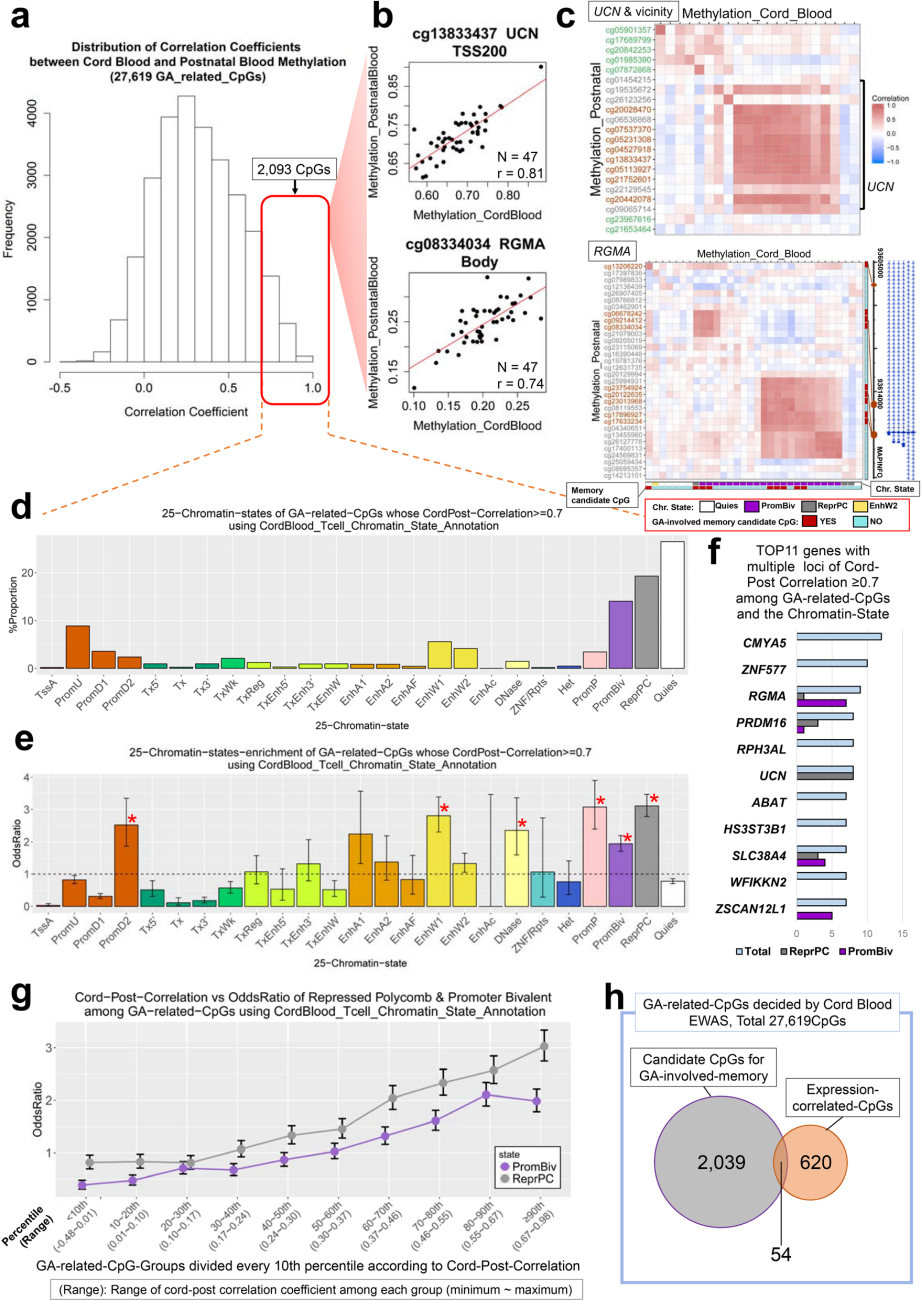
Candidate CpGs for GA-involved epigenetic memory whose methylation alteration persist after birth. (**a**) Histogram showing distribution of correlation coefficients between cord blood and postnatal blood methylation levels among the 27,619 GA-related CpGs decided by the cord blood EWAS (n = 110). Red block denotes the 2,093 CpGs of correlation coefficients ≥ 0.7 (n = 47), *i.e.*, candidate CpGs whose methylation alteration persist after birth. (**b**) Scatterplots indicating cord blood methylation level (x-axis) versus postnatal blood methylation level for 2 candidate CpGs. These CpGs existed in the representative genes with multiple candidate CpGs in chromatin state of ‘Repressed Polycomb’ or ‘Bivalent Promoter’. (**c**) Heatmap showing Pearson correlation between cord blood and postnatal blood CpG methylation levels in 2 regions (*UCN* with its neighborhood (top panel), the midst of *RGMA* (bottom panel)). Each column (row) represents a cord (postnatal) blood CpG methylation level. Row labels indicate CpG names, and a dark red label means a candidate CpG for epigenetic memory, and a green label means a CpG nearby *UCN*. (**d**) Distribution of 25 chromatin states for GA-related CpGs whose cord-post correlation ≥ 0.7 using cord blood T cell-based annotation of ChromHMM. (**e**) Enrichment of 25 chromatin states for GA-related CpGs whose cord post-correlation ≥ 0.7. Single red asterisk denotes the enriched chromatin state which was significant at Bonferroni-criteria (0.05/25) and of enrichment odds ratio ≥ 1 (black dashed line). (**f)** Top 11 genes with multiple loci of cord-post correlation ≥ 0.7 among GA-related CpGs and those CpGs’ chromatin state. (**g**) A line graph showing the relationship between the cord-post correlation level of methylation (x-axis) and the enrichment odds ratio of 2 chromatin states (‘Repressed Polycomb’, ‘Bivalent Promoter’). 27,619 GA-related CpGs were divided into 10 bins every 10th percentile for cord-post correlation coefficients. (**h**) Overlap between 2093 candidate CpGs for GA-involved epigenetic memory (gray circle) and 674 CpGs whose methylation correlated with log_2_-transformed expression of corresponding genes (orange circle: see Fig. 4) among GA-related CpGs. *GA: gestational age, *EWAS*: epigenome-wide association study. **All error bars indicate 95% CI (confidence interval). ***Chromatin state abbreviations are defined in ChromHMM. Following Abbreviations are defined in ChromHMM; TssA: Active TSS, PromU: Promoter upstream TSS, PromD1: Promoter downstream with DNase, PromD2: Promoter downstream TSS, Tx5′: Transcription 5′, Tx: Transcription, Tx3′: Transcription 3′, TxWk: Weak Transcription, TxReg: Transcription Regulatory, TxEnh5′: Transcription 5′ Enhancer, TxEnh3′: Transcription 3′ Enhancer, TxEnhW: Transcription Weak Enhancer, EnhA1: Active Enhancer 1, EnhA2: Active Enhancer 2, EnhAF: Active Enhancer Flank, EnhW1: Weak Enhancer 1, EnhW2: Weak Enhancer 2, EnhAc: Enhancer Acetylation Only, DNase: DNase only, ZNF/Rpts: ZNF genes & repeats, Het: Heterochromatin, PromP: Poised Promoter, PromBiv: Bivalent Promoter, ReprPC: Repressed Polycomb, Qies: Quiescent/Low.

Next, we examined the relationship between DNA methylation at birth and methylation postnatally around the expected due date. Pearson’s correlation coefficients between 47 paired cord and postnatal peripheral blood mononuclear cell methylation values were calculated for all 27,619 GA-related CpGs identified in the first EWAS of 110 cord blood samples. The median time interval between the two blood draws was 7.1 weeks (range: 2.0 to 18.1 weeks). We considered CpGs of correlation coefficient ≥ 0.7 as the candidates for GA-involved epigenetic memory, similar to definitions used in previous reports^19^. We identified 2,093 candidate CpGs for GA-involved epigenetic memory that showed a correlation coefficient ≥ 0.7 (Fig. 5a; Supplementary Table 18). Among these, CpGs showing high methylation at birth were likely to remain high after the birth as well, while those of low methylation values at birth appeared to remain low after birth (Fig. 5b). To evaluate the possibility that the correlation coefficients may be influenced by the time interval of the two blood draws, we ordered the samples by time interval and compared the correlation coefficients of the bottom 23 samples (median time interval: 4.0 weeks) and top bottom 23 samples (median time interval: 11.0 weeks) by using paired *t*-tests. The mean of the bottom group was only 0.036 (95%CI: (0.030, 0.042)) higher than that of the top group (Supplementary Fig. 12), and the distribution of correlation coefficients of the two groups were similar. High correlations were observed across multiple CpGs in genes such as *UCN* and *RGMA*. The methylation of certain CpGs in these genes were also correlated with neighboring CpGs in cord and postnatal blood samples. This may indicate that specific genomic regions were regulated in the same way during the perinatal period (Fig. 5c). To characterize those regions, we referred to ChromHMM 25-chromatin-states of the 2,093 candidate CpGs for GA-involved epigenetic memory. Among these 2,093 CpGs studied, “Repressed Polycomb” and “Bivalent Promoter” showed as the second and the third highest frequency after “Quiescent/Low” in both cord blood T cell-based and B cell-based annotations (Fig. 5d; Supplementary Fig. 13, Supplementary Table 19). These two chromatin states were also enriched in Fisher’s exact test in both annotations, and “Repressed Polycomb” was most significantly enriched (Fig. 5e). Further, as the correlation coefficients between cord blood and postnatal blood DNA methylation increased, the proportion of loci occupied by “Repressed Polycomb” or “Bivalent Promoter” increased as well (Fig. 5g). Indeed, CpGs in *UCN* and *RGMA*, where the DNA methylation levels of each CpG correlated well with neighboring CpGs, were mainly in a state of “Repressed Polycomb” and “Bivalent Promoter”, respectively (Figs. 4f, 5c). Apart from these two genes, multiple CpGs in *PRDM16*, *SLC38A4*, and *ZSCAN12L1*, which were included in the 2,093 candidate CpGs for GA-involved epigenetic memory, were also in a state of “Repressed Polycomb” and/or “Bivalent Promoter” (Fig. 5f).

Finally, we investigated transcription of the aforementioned candidate CpGs for GA-involved epigenetic memory. Of the 2,093 candidate CpGs, 54 had methylation expression correlation in cord blood (Fig. 5h), where half of the CpGs had a negative correlation and the other half had a positive correlation (Supplementary Table 20). From these 54 CpGs, 8 CpGs located in *UCN* were identical to the ones in the multiple ‘discordant’ promoter CpGs of Fig. 4e. Thus, cord blood DNA methylation of 8 CpGs in the *UCN* promoter were GA-related, positively correlated with expression, and also correlated with own postnatal blood methylation. However, 97.4% of the 2,093 candidate CpGs showed no methylation expression correlation at birth in cord blood.

## Discussion

In this study, 27,619 GA-related CpGs and 150 SD score-related CpGs were initially identified from the cord blood EWAS. Secondly, 461 GA-related transcripts and no SD score-related transcripts were found, and methylation expression correlations among approximately two-thirds of GA-related CpG transcript combinations were observed. Lastly, 2093 candidate CpGs for GA-involved epigenetic memory were identified. Among these candidates, trends of chromatin states, such as, “Repressed Polycomb” was observed, alongside the confirmation of 54 CpG correlations with transcription, and the non-negligible number of discordant CpGs where transcription increased as methylation increased.

Four studies have previously been conducted on GA-related CpGs and/or GA-prediction CpGs. More than 75% of the GA-related CpGs reported by Schroeder et al. (41 CpGs identified in a discovery cohort, 26 of which replicated^13^) or ARIES Cohort (224 CpGs^15^) were also identified in our study. On the other hand, only approximately 40% of our 27,619 GA-related CpGs were found among the 44,359 CpGs associated with ultrasonography-determined GA (Bohlin et al.) within the MoBa Cohort data^17^ (Supplementary Fig. 14). However, regarding RefSeq genes within 250 bp upstream or downstream to CpGs, approximately 80% of our GA-related RefSeq genes were common to the Bohlin et al. study. The discrepancy of CpG loci between the present study and Bohlin et al. study may be attributed to racial differences (Japanese vs Norwegian) and/or sampling methods used (mononuclear cell separation (lymphocyte-dominant) vs buffy coat without additional cell isolation (granulocyte-dominant)). Of the 131 CpGs for GA-prediction identified by Bohlin et al.^17^, 108 were found among our GA-related CpGs while 50 of 148 CpGs reported by Knight et al.^16^ (using the method developed by Horvath^26^) were observed in our study (Supplementary Fig. 15). The difference in overlap between studies may be attributed to the selection methods of GA-prediction CpGs. In contrast, our transcription-correlated GA-related CpGs or epigenetic memory candidate CpGs overlapped only minimally with the aforementioned prediction CpGs. Thus, most epigenetic memory candidate CpGs may not be suitable for predicting accurate GA, which may be reasonable based on the understanding that memory methylation would not undergo changes *ex utero* according to chronological time passing.

The CpGs associated with birth weight in previous studies (Engel et al., MoBa; Simpkin et al., ARIES)^15,18^ or CpGs associated with “birth weight SD score for GA”^19^ were not observed among birth weight SD score-related CpGs in our study. One reason for such discrepancies may lie with this study’s participant population whose ratio of preterm to term infants was approximately 1.5, whereas the previous studies pertained mainly to term infants. Second, the results may be affected by differences in nutritional and environmental status of mothers between the study areas. For instance, Japanese mothers are likely to have less body-weight gain during pregnancy than mothers in other countries^27^.

There are several strengths to note compared with previous cord blood EWAS of GA. Firstly, we conducted an integrative analysis of the methylome and simultaneously generated transcription data. Secondly, we evaluated methylation persistence between samples at birth and samples at the expected due date, and among the CpGs of persistent methylation we discovered novel trends in chromatin state and transcription.

Although there have been previous reports on GA-related CpGs, we also identified 461 GA-related transcripts by using the same samples as those used in the EWAS of GA. In addition, the NF-kappa B signaling pathway was enriched in both negatively GA-related CpGs and positively GA-related transcripts while the PI3K-Akt signaling pathway was enriched in the inverted manner. These relationships are consistent with the accepted notion that higher methylation in promoter region leads to repressed gene expression, thereby supporting the validity of our analyses. Correlations between methylation and transcription were confirmed in approximately 2/3 of GA-related transcripts. Among these correlated CpG-transcript combinations, the combinations of positive correlations were found in approximately half as many as negatively correlated combinations – a non-negligible number. In the promoter region, positive correlation between methylation and transcription means a discordant CpG-transcript relation where transcription increases as methylation increases. Recent studies targeting other sample types revealed that CpGs of such discordant relation are not uncommon^24,28^. In the present study, “Repressed Polycomb” of the 25 chromatin states in ChromHMM had the highest number among discordant promoter CpGs. It was also reported that the relation between methylation and transcription at the polycomb-binding site could be opposite from that of other sites^29,30^.

With respect to GA-involved epigenetic memory, we identified 2,093 candidate CpGs based on a correlation coefficient ≥ 0.7 between cord and postnatal blood methylation, although we did not identify any FDR-significant CpGs in the postnatal blood EWAS whose methylation was associated with GA at birth. These results suggest that some epigenetic effects of preterm birth may tend to persist postnatally, but there may be fluctuations in the degree of epigenetic memory. Also, methodologically, the lack of significant CpGs may have been due to limited statistical power. Furthermore, we demonstrated that the states of “Repressed Polycomb” and “Bivalent Promoter” were enriched among the GA-related epigenetic candidate CpGs. These two types of chromatin states are based on the repressive histone mark of H3K27me3^25^, or related to polycomb-binding, and are closely connected^31^. Polycomb-binding, or histone modification of H3K27me3, is involved in epigenetic memory in plants^32,33^. GA-involved epigenetic memory candidate CpGs did not necessarily show relationship between methylation and corresponding transcription, with only 54 candidate CpGs found to have significant correlation with gene expression. There were also 11 discordant promoter CpGs among these 54 sites (Supplementary Table 20). *UCN*, *SLC12A7*, *TNFAIP2*, *ANGPT2*, and *NGF* were the only genes within 250 bp upstream or downstream of regions that contained ≥ 3 CpGs and showed an association with GA, correlation with transcription, and correlation coefficient ≥ 0.7 between birth and at around due date. *UCN* showed the largest number of significant CpGs. The EWAS by Bohlin et al. identified multiple GA-related CpGs of *UCN*, *SLC12A7*, and *NGF* by using MoBa cohort data^17^, and among them cg20442078 and cg05231308 of *UCN* were also found in our list of transcription-correlated GA-involved epigenetic memory candidates (Supplementary Table 20). *UCN* encodes Urocortin, an endogenous peptide hormone belonging to the corticotropin-releasing hormone (CRH) family^34^. Urocortin is found in the central nervous system (CNS) and peripheral tissues such as the heart, adrenal glands, and lymphocytes. Urocortin influences stress responses in the CNS, cardiovascular system and immune system via CRH receptors 1 or 2. For instance, it has protective effects against myocardial ischemic reperfusion injury^35^. All the loci of *UCN* presented ‘discordant’ methylation-expression correlations with all these CpGs annotated as “Repressed Polycomb” among the 25 chromatin states. This finding may be possible as discordance has been observed for polycomb-binding sites as described previously. *UCN* was one of the top two genes that had multiple candidate CpGs for GA-involved epigenetic memory with the modification of H3K27me3; the other top gene was *RGMA* whose major chromatin state was ‘Bivalent Promoter’. *RGMA* encodes repulsive guidance molecule A, a potent inhibitor of nerve growth that is expressed in several brain diseases, including Alzheimer’s disease and multiple sclerosis^36^. *RGMA* has also been reported to play an inhibitory role in cancer progression^37^. Further, a recent study has suggested that *RGMA* may have an important role in the communication between the sympathetic nervous system and inflammation via monocyte activation through the suppression of NF-κB activity and activation of PI3K-Akt-signaling^38^.

It is important to acknowledge certain limitations of this study. The sample size was relatively small and varied across the different analyses, and we were not able to attempt replication in an independent cohort. This was largely due to the trade-off between sample size and experimental effort including mononuclear cell isolation and simultaneous DNA/RNA extraction. Despite the modest sample size, we confirmed the same GA-related CpGs found in previous EWAS, identified novel candidates, and were also able to integrate the EWAS findings with transcription data. In the future, it is desirable to evaluate DNA methylation and gene expression simultaneously by using samples from larger populations with plans for replication attempts. Second, the samples for evaluation of epigenetic memory were obtained twice within a short interval between birth and expected due date. A previous study reported that most methylation alteration associated with GA disappears by the age of 7 years^15^. On the other hand, this study successfully demonstrated for the first time that most of these associations were attenuated by around the due date while some epigenetic effects in preterm infants tend to persist at least for several weeks to months. Thus, we consider the use of our postnatally collected samples to be informative in the assessment of epigenetic memory focused on the early life period.

In conclusion, integrative analysis of cord blood methylome and transcription data identified many GA-related methylation alterations and some related to birth weight SD score. We subsequently confirmed methylation expression correlations among candidate CpGs whose methylation was associated with GA. We also identified methylation alterations generated in preterm birth that persisted after birth, thus suggesting GA-involved epigenetic memory. Among these candidate CpGs for epigenetic memory, we found trends of chromatin state such as repressed polycomb-binding sites.

## Methods

### Ethics statement

All methods were carried out in accordance with following ethical guidelines in Japan: Ethics Guidelines for Human Genome/Gene Analysis Research, Ethical Guidelines for Medical and Health Research Involving Human Subjects, Ethical Guidelines for Epidemiological Research. Our study was approved by the following institutional ethics committees: Human Genome Ethics Committee of The University of Tokyo Hospital (approval ID: G10036); Ethics Committee of National Center for Child Health and Development (approval ID: 234); Ethics and Personal Information Protection Committee of Tokyo Metropolitan Bokutoh Hospital (approval ID: 38). All participant mothers provided written informed consents for themselves and their infants.

### Study population

We implemented a cross-sectional study design with prospective recruitment of mother-infant pairs considered eligible if they were East Asian, had a live birth at 22–42 weeks gestation, and no fetal congenital disease prenatally diagnosed. Recruitment was targeted for more than 100 mother-infant pairs (median sample size was 96 based on four major previous studies^13,14,20,39^). Between October 2014 and July 2016, 147 mother-infant pairs were invited to participate around the time of delivery at the University of Tokyo Hospital or at the Tokyo Metropolitan Bokutoh Hospital, among which 144 mothers provided written informed consents, and 3 refused participation (Supplementary Fig. 1). Umbilical cord blood samples were obtained from all participants at the time of infant delivery, and postnatal peripheral blood samples were obtained by venipuncture at least 2 weeks after birth around their due date (36–44 weeks of postmenstrual age). Postnatal blood samples were unavailable for 74 participants for various reasons, but primarily due to hospital discharge occurring prior to 2 weeks after birth (Supplementary Fig. 1).

### Covariates

Clinical data (*e.g.* GA, birth weight, mode of delivery, etc*.*), maternal age and pre-pregnancy BMI, use of assisted reproductive technology (*e.g. *in vitro fertilization (IVF), etc.), mother’s smoking status and pregnancy complications, as well as postmenstrual age during postnatal blood collection were obtained from hospital medical records. Paternal data (*i.e.* body weight, height, age) were obtained by questionnaire. Birth weight SD score – namely birth weight z-score for GA according to Japanese reference data – was calculated from GA, birth weight, infants’ sex, and parity, through a program provided by the Japanese Society for Pediatric Endocrinology (downloaded from following site on 16th, December, 2016; http://jspe.umin.jp/medical/keisan.html).

### Mononuclear cell separation and DNA/RNA extraction

From the obtained blood samples, cord blood mononuclear cells (CBMCs) and peripheral blood mononuclear cell (PBMC) samples were separated by gradient centrifugation using Ficoll-Hypaque within 24 h of sample collection (details are described in Supplementary Fig. 3). The buffy coats of CBMCs/PBMCs were directly lysed with Buffer RLT Plus (Qiagen) containing β-mercaptoethanol in 59 cord blood samples and 14 postnatal blood samples. Genomic DNA was extracted from the lysate of mononuclear cell buffy coat using Qiagen AllPrep kit (Qiagen) (Protocol 1). In the remaining 76 cord blood samples and 52 postnatal blood samples, we utilized Protocol 2, in which the CBMCs/PBMCs buffy coats were processed with erythrocyte lysis solution, and genomic DNA and total RNA were simultaneously extracted from the lysate of the isolated cells using the same kit in Protocol 1. Aliquots of genomic DNA and total RNA were then stored at − 80 °C.

### Quality control (QC) and preprocessing in DNA methylation microarray analysis

Following extraction, genomic DNA was bisulfite-converted using the EpiTect Plus DNA Bisulfite Kit (Qiagen). Bisulfited DNA was processed with 450 k array, which can assay methylation levels at more than 485,000 CpG sites. The methylation levels at each CpG site was calculated as β values. The methylation levels at each CpG site was calculated as β values where β  = intensity of the methylated allele (*M*) / (intensity of the unmethylated allele (*U*) + intensity of the methylated allele (*M*) + 100). Therefore, β values ranged from 0 (completely unmethylated) to 1 (completely methylated). All methylation data preprocessing was conducted in R environment (v. 3.3.2). The quality of each sample was evaluated using RnBeads package (v. 1.4.0)^40^ and *minfi* package (v. 1.20.2)^41^. Failed samples were excluded on the basis of bisulfite conversion efficiency, hybridization efficiency, and the intensity of methylated and unmethylated probes.

After exclusion of low-quality samples, CpG probes were filtered using the ChAMP package (v. 2.6.0)^42^. Non-CpG probes were removed. The probes on the X and Y chromosomes, with a detection *p* value > 0.01, and a beadcount less than 3 were removed. Based on the default of the ChAMP package, the probes mapping to multiple sites, defined by Nordlund et al*.*^43^, were removed. In addition, according to the data of Chen et al*.*^44^, cross-reactive and polymorphic CpG probes of Asian minor-allele frequency (MAF) ≥ 1% were also removed. After filtering, 410,735 CpG probes remained for further analysis. Background correction and dye-bias equalization (Noob)^45^ was then performed using the *minfi* package. To further reduce the bias of type 2 probe values, beta-mixture quantile normalization (BMIQ)^46^ was performed using the ChAMP package.

### Estimation of cell fraction

Cell fraction was estimated with the method proposed by Houseman^47,48^ using the Bakulski reference data set for cord blood analysis^49^. Estimated cell fraction data including lymphocytes (CD4 + T cells, CD8 + T cells, NK cells, B cells), myelocytes (granulocytes, monocytes), and nucleated red blood cells (nRBCs) were used for further multivariate methylation analysis.

### Gene expression microarray analysis and data preprocessing

Following extraction, total RNA was initially quantified and qualitatively assessed whereby samples with low RNA yield and quality were excluded (Supplementary Fig. 4). Thereafter, 100 ng of total RNA was used to produce Cyanine 3-labeled cRNA. After labeling, 600 ng of cRNA was fragmented and hybridized to the SurePrint G3 Human GE microarray 8 × 60 K Ver. 3.0 (Agilent Technologies). After hybridization, washing the array slides, and scanning, the raw intensity data were obtained using Agilent Feature Extraction (FE) software (ver. 10.7.3.1).

As described in Supplementary Fig. 4, array QC on each sample, probe filtering, and normalization were performed using GeneSpring14.5 (Agilent Technologies). The data set comprised of 65 cord blood and 48 postnatal peripheral blood samples that passed QC; these were then quantile normalized with the minimum expression level of all transcription among all samples set to 1. Probes above the threshold expression criteria were selected, and those on sex chromosomes were removed. Finally, 46,789 probes remained for further analysis. After this preprocessing, the samples from twin infants were excluded. As the number of postnatal blood RNA samples were small, these were not used for further analysis. In total, 55 cord blood RNA samples remained. Prior to transcription analysis, expression probes were selected from among the 46,789 remaining probes according to the results of the cord blood EWAS; probes without threshold background signal detection were removed. The normalized and filtered data were imported into the R environment for statistical analysis.

### Statistical analysis

All statistical analysis was conducted in the R environment (v. 3.3.2). The overall analysis framework is summarized in Supplementary Fig. 5, and the detail of each analysis is described below.

#### Covariates associating with GA and/or birth weight SD scores

Simple linear regression analysis was performed to examine the association between prenatal variables and infant GA and birth weight SD scores. Infant sex and six prenatal variables associated with GA and/or birth weight SD scores were included in subsequent analyses. These variables were subject to mutual statistical adjustment in multivariate linear regression analysis of GA and SD scores. As described by Gelman^50^ and Lin et al.^51^, binary prenatal variables were not scaled, and continuous prenatal variables were standardized to have a standard deviation of 0.5 in order to compare effect estimates from both continuous and binary prenatal variables.

#### Epigenome-wide association analysis of GA and/or birth weight SD scores and pathway analysis

Two multivariate linear regression models were used to evaluate the association between GA and SD scores and cord blood DNA methylation value for each CpG site.

$$ Y_{{meth}} ~ = {\text{~}}\beta _{0} {\text{~}} + {\text{~}}\beta _{{GA}} {\text{~}} \times {\text{~}}X_{{GA}} {\text{~}} + {\text{~}}\beta _{{SDscore}} {\text{~}} \times {\text{~}}X_{{SDscore}} {\text{~}} + {\text{~}}\sum \left( {\beta _{i} ~ \times ~X_{{COV}} } \right)~ + ~\varepsilon $$

The two linear regression models differed in covariate adjustment, in which the first model (Model 1) adjusted for infant sex, batch and estimated cellular populations, and the other model (Model 2) additionally adjusted for the above mentioned 6 prenatal covariates associated with GA and/or SD score. To adjust for multiple testing across 410,735 probes, suggestive CpG sites associated with GA or birth weight SD scores were selected at an FDR of < 0.05 in each model. Finally, we defined GA-related CpGs or SD score-related CpGs as CpG sites whose methylation levels were associated with GA or SD scores in both “Model 1” and “Model 2” in the same direction.

In preparations for pathway analyses, we categorized the suggestive CpGs based on the direction of regression coefficients (*i.e.* positive or negative) for both the analysis of GA and SD score, giving rise to four categories of CpGs. Next, the four categories of CpGs were linked to genes using the ChAMP package^23^. In addition to duplicate gene entities, probes lacking an Illumina gene annotation, and probes mapped to gene body, 3′UTR, or intergenic region were not used for pathway analyses. Among the gene entities of each CpG category, only entities with at least 2 CpGs on them were selected. Finally, DAVID Bioinformatics Resources 6.8^23^ was used to assess enrichment in KEGG pathway, and Benjamini–Hochberg procedure was applied to this analysis based on the FDR; an enrichment-FDR threshold of ≤ 0.1 was used based on the default applied by the DAVID resource (https://david.ncifcrf.gov/content.jsp?file=functional_annotation.html).

## Supplementary Information

Supplementary Information 1.

Supplementary Information 2.

## Data Availability

Both the methylation microarray data and the gene expression microarray data have been deposited in the Gene Expression Omnibus (GEO) database under accession number GSE110829.
