# Supplementary material for: Identification of epigenetic memory candidates associated with gestational age at birth through analysis of methylome and transcriptional data
Source: Sci Rep. 2021 Feb 9;11:3381. doi: 10.1038/s41598-021-83016-3 (PMC7873311; doi:10.1038/s41598-021-83016-3)
Supplement: Supplementary file 1 — Supplementary Information 1. [file 41598_2021_83016_MOESM1_ESM.pdf]

Title: **Supplementary Information − Identification of epigenetic memory candidates associated with gestational age at birth through analysis of methylome and transcriptional data**

[1, 2, 14, *]Kohei Kashima, [2, 14]Tomoko Kawai, [1]Riki Nishimura, [3]Yuh Shiwa, [4, 5]Kevin Y Urayama, [2]Hiromi Kamura, [6]Kazue Takeda, [7]Saki Aoto, [1]Atsushi Ito, [8]Keiko Matsubara, [9]Takeshi Nagamatsu, [9]Tomoyuki Fujii, [10]Isaku Omori, [10]Mitsumasa Shimizu, [11]Hironobu Hyodo, [11]Koji Kugu, [6]Kenji Matsumoto, [3, 12]Atsushi Shimizu, [1]Akira Oka, [13]Masashi Mizuguchi, [2]Kazuhiko Nakabayashi, [2]Kenichiro Hata, [1]Naoto Takahashi

1 Department of Pediatrics, The University of Tokyo Hospital, Tokyo, Japan

2 Department of Maternal-Fetal Biology, National Research Institute for Child Health and Development, Tokyo, Japan

3 Division of Biomedical Information Analysis, Iwate Tohoku Medical Megabank Organization, Disaster Reconstruction Center, Iwate Medical University, Iwate, Japan

4 Department of Social Medicine, National Research Institute for Child Health and Development, Tokyo, Japan

5 Graduate School of Public Health, St. Luke's International University, Tokyo, Japan

6 Department of Allergy and Clinical Immunology, National Research Institute for Child Health and Development, Tokyo, Japan

7 Medical Genome Center, National Research Institute for Child Health and Development, Tokyo, Japan

8 Department of Molecular Endocrinology, National Research Institute for Child Health and Development, Tokyo, Japan

9 Department of Obstetrics and Gynecology, The University of Tokyo Hospital, Tokyo, Japan

10 Department of Neonatology, Tokyo Metropolitan Bokutoh Hospital, Tokyo, Japan

11 Department of Obstetrics and Gynecology, Tokyo Metropolitan Bokutoh Hospital, Tokyo, Japan

12 Division of Biomedical Information Analysis, Institute for Biomedical Sciences, Iwate Medical University, Iwate, Japan

13 Department of Developmental Medical Sciences, The University of Tokyo, Tokyo, Japan

14 These authors contributed equally: Kohei Kashima and Tomoko Kawai.

Corresponding Author: Kohei Kashima

Department of Pediatrics, The University of Tokyo Hospital

Hongo, Bunkyo-ku, Tokyo, 113-8655, Japan

Phone: +81-3-5800-8659 / Fax: +81-3-3816-4108

E-mail: KASHIMAK-PED@h.u-tokyo.ac.jp

Contents

Supplementary Methods

Supplementary Figure: **1~15**

Supplementary Table: 1~5, 8~10, 13, 14, 16, 17, 19, 20; These tables are provided in this file.

Supplementary Table 6, 7, 11, 12, 15, 18: These tables are provided in Excel spreadsheet.

Supplementary Methods

Study population (filtering)

For cord blood DNA methylation microarray analysis, among the 144 cord blood samples, we excluded those that had low estimated lymphocytes < 5×10^5 (n = 5); were of low quality (n = 7); obtained from participants shown to have congenital diseases (n = 4); and a sample obtained from an egg-donor offspring (n = 1). Additionally, we excluded samples from twin infants (n = 17) subsequent to microarray data normalization

(Supplementary Fig. 1). For postnatal blood methylation analysis, among the 70 postnatal blood samples, we excluded sample outliers based on postmenstrual age (n = 4), from twin infants (n = 14) and infants without valid cord blood methylation data (n = 5). The final analytic number for cord blood DNA methylation analysis was 110, and a subset of 47 infants were available for postnatal peripheral blood DNA methylation analysis.

For cord blood expression microarray analysis, we excluded samples with low estimated lymphocytes < 5×10^5 (n = 5); without the use of erythrocyte lysing buffer use in mononuclear cell isolation (n = 59); of low quality (n = 9); and samples with no valid DNA methylation data (n = 6). Additionally, we excluded twin infants (n = 10) resulting in a final analytic number of 55 samples for cord blood RNA expression analysis (Supplementary Fig. 2). Lastly, of the 70 postnatal blood samples available, 35 were excluded for various reasons (Supplementary Fig. 2); due to the small numbers, postnatal peripheral blood RNA expression analysis was not performed.

Statistical analysis

Association analysis of transcription and GA and/or birth weight SD scores and pathway analysis. To explore the gene expression changes associated with GA and SD score, we performed association analysis focused on the genes that showed methylation

associations with GA and/or SD score in the cord blood EWAS. Among the 27,701 FDR-significant CpGs associated with GA and/or SD score, we matched 15,038 CpGs to 7,369 QC-filtered gene expression probes within a region of 250 kb upstream or downstream of the CpG by using the Homer annotation tool[1]. Multivariate linear regression analysis was used to evaluate the association between GA and SD score and log_2-transformed cord blood gene expression value for each probe (same covariates as Model 1 in the cord blood EWAS). To adjust for multiple testing across 7,369 probes, gene expression probes associated with GA or SD scores were selected at an FDR of < 0.05[2].

After association analysis of transcription and GA and SD scores, we conducted pathway analysis for FDR-significant probes, and subsequently performed the same analysis for nominally significant probes (nominal p-value < 0.05). We categorized the associated transcripts based on the directionality of regression coefficients. Probes lacking an Agilent gene annotation were not considered in the pathway analyses, as well as duplicate gene entities. Finally, DAVID Bioinformatics Resources 6.8[3] was used to analyze enrichment in KEGG pathway. Benjamini-Hochberg procedure was applied to these analyses based on the FDR; an enrichment-FDR threshold of ≤ 0.1 was used based on the default of the DAVID resource (https://david.ncifcrf.gov/content.jsp?file=functional_annotation.html).

Methylation expression analysis. To confirm the direct association between DNA methylation and gene expression, we performed *cis*-association analysis targeting the gene loci that had both FDR-significant CpGs and transcripts related to GA. We generated 1,355 CpG-transcript combinations composed of 461 GA-related transcripts derived from 414 RefSeq genes and 1,196 corresponding GA-related CpGs by using the Homer annotation tool[1] (Supplementary Fig. 9a). The associations were examined using linear regression, and a nominal p-value < 0.05 was considered statistically significant.

Identification of candidate CpGs whose methylation alteration persist after birth. Linear regression analysis was used to evaluate the association between GA and cord blood and postnatal peripheral blood methylation among 47 infants who had valid methylation data. Next, we selected candidate CpGs based on the results of the cord blood EWAS of GA (n = 110) and examined the correlation (Pearson's correlation coefficient) between cord blood and postnatal peripheral blood methylation for GA-related CpGs. We considered CpGs of correlation coefficient ≥ 0.7 as the candidates for GA-involved epigenetic memory based on previous reports[4]. In addition, we conducted enrichment analysis of the 25 chromatin states that were characterized by 12 modifying marks in

ChromHMM[5]. In this enrichment analysis, we utilized cord blood T cell-based and cord blood B cell-based chromatin-states' annotation. Fisher's exact test was used to analyze enrichment in the 25 chromatin states. Bonferroni procedure was applied for multiple testing adjustment; a threshold of Bonferroni-adjusted-p-value (nominal p-value $\times$ 25) < 0.05 was used.

Supplementary Figures

1: Study inclusion & DNA methylation sample filtering

2: Flow of RNA sample filtering

3: Mononuclear cell separation and DNA/RNA extraction

4: Gene expression microarray analysis and data preprocessing

5: Overall Analysis Framework

6: Manhattan Plot of p-values for associations between DNA methylation and gestational age

7: Manhattan Plot of p-values for associations between DNA methylation and birthweight SD score

8: Distribution of CpGs associated with gestational age and/or birth weight SD scores

9: Association between GA-related transcription and corresponding GA-related CpG

methylation

10: Top 8 genes that have multiple GA-related CpGs of ‘concordant’ methylation expression relation

11: Association of gestational age with DNA methylation at birth and on a postnatal day around the expected due date

12: Distribution of cord blood and postnatal sample (cord-post) correlation coefficients of two groups selected according to the time interval between the two blood draws for 2093 candidate CpGs for GA-involved epigenetic memory

13: Chromatin states evaluation among candidate CpGs for GA-involved epigenetic memory by using cord blood B cell-based annotation in ChromHMM

14: Comparison with the GA-related CpGs identified in previous EWAS

15: Comparison with the CpGs which were selected for the purpose of predicting gestational age utilizing their methylation data

<u>Supplementary Tables</u>

1: Pregnancy- and delivery-related characteristics of 110 mother-infant pairs – Complement of “Table 1”

2: Association of prenatal covariates with gestational age – Result of univariate linear

regression analysis

3: Association of prenatal covariates with birth weight SD scores – Result of univariate linear regression analysis

4: Association of prenatal covariates with gestational age – Result of multivariate linear regression analysis

5: Association of prenatal covariates with birth weight SD scores – Result of multivariate linear regression analysis

6: Result of cord blood EWAS on GA using 110 samples; GA-related CpGs (27,619 sites) (Excel Spreadsheet)

7: Result of cord blood EWAS on birth weight SD scores using 110 samples; birth weight SD score-related CpGs (150 sites) (Excel Spreadsheet)

8: Confirmation of the results of 27,619 GA-related CpGs based on "Model 1" and "Model 2" − Comparison with the results of sensitivity analysis by using Model 1 adjusted for each of the 6 prenatal covariates of "Model 2"

9: Confirmation of the results of 150 SD score-related CpGs based on "Model 1" and "Model 2" − Comparison with the results of sensitivity analysis by using Model 1 adjusted for each of the 6 prenatal covariates of "Model 2"

10: Enrichment in KEGG pathway for the GA-related CpGs

11: Result of transcription association analysis on GA using 55 cord blood samples; GA-related transcripts (1,611 transcripts selected at the criteria of nominal-p <0.05) (Excel Spreadsheet)

12: Result of transcription association analysis on birth weight SD scores using 55 cord blood samples (6 transcripts selected at the criteria of nominal-p <0.05) (Excel Spreadsheet)

13: Enrichment in KEGG pathway for the GA-related expression probes selected based on the criteria of FDR

14: Enrichment in KEGG pathway for the GA-related expression probes selected based on the criteria of nominal p-value

15: Result of methylation expression analysis using 55 cord blood samples; Correlation between % methylation and $\log_2$-transformed expression; 757 combinations selected at the criteria of nominal-p <0.05 (Excel Spreadsheet)

16: Distribution and enrichment of the 25 chromatin states among 'discordant' GA-related CpGs that had positive correlation between methylation and corresponding $\log_2$-transformed transcription using cord blood T cell-based annotation provided by ChromHMM

17: Distribution and enrichment of the 25 chromatin states among 'concordant' GA-

related CpGs that had positive correlation between methylation and corresponding log_2-transformed transcription using cord blood T cell-based annotation provided by ChromHMM

18: Candidate CpGs for GA-involved epigenetic memory by the analysis using 47 babies who provided both cord blood and postnatal blood data; 2,093 CpGs with cord post correlation ≥ 0.7 (Excel Spreadsheet)

19: Enrichment of the 25 chromatin states among 2,093 candidate CpGs for GA-involved epigenetic memory in the analysis using cord blood T cell-based and B cell-based annotation provided by ChromHMM

20: Transcription-correlated & GA-involved epigenetic memory candidate CpGs

Supplementary Figure 1

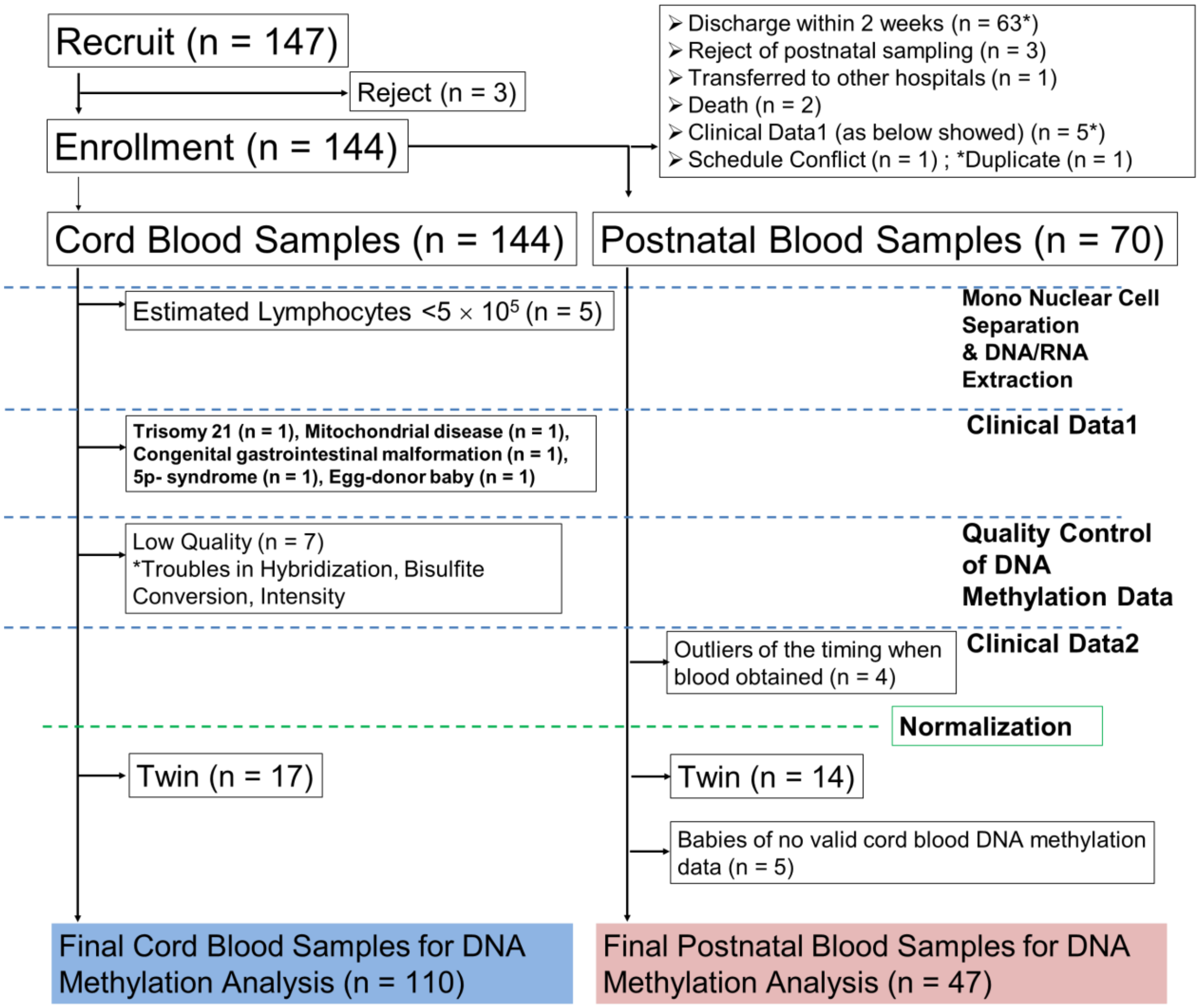

Supplementary Figure 1. Study inclusion & DNA methylation sample filtering. Around the time of deliveries, 147 mother-infant pairs were recruited, and 144 pairs provided written informed consents. Cord blood samples were obtained from all participants, and postnatal peripheral blood samples were obtained 2 weeks or later after birth only when the babies' physical condition was stable past 36 weeks of postmenstrual age but in hospital. Postnatal blood samples were unavailable from following participants; babies who discharged within 2 weeks after birth (n = 63), whose parents rejected of postnatal sampling (n = 3), who were transferred to other hospitals (n = 1), who died before postnatal sampling (n = 2), who proved to be congenital diseases (n = 4), and who had schedule conflicts (n = 1). Subsequently, 70 postnatal blood samples were available. For cord blood DNA methylation microarray analysis, from the obtained 144 cord blood samples, we excluded the samples that had low estimated lymphocytes $<5 \times 10^5$ (n = 5), were of low quality (n = 7), and obtained from the participants proved to be trisomy 21

(n = 1), mitochondrial disease (n = 1), congenital gastrointestinal malformation (n = 1), 5p- syndrome (n = 1), and egg-donor baby (n = 1). Additionally, we eliminated the samples from twin (n = 17) after microarray data normalization finished. For postnatal blood methylation analysis, From the obtained 70 postnatal blood samples, we excluded sample outliers based on postmenstrual age (n = 4). In addition, we eliminated samples from twins (n = 14) and babies without valid cord blood methylation data (n = 5) after microarray data normalization finished.

Supplementary Figure 2

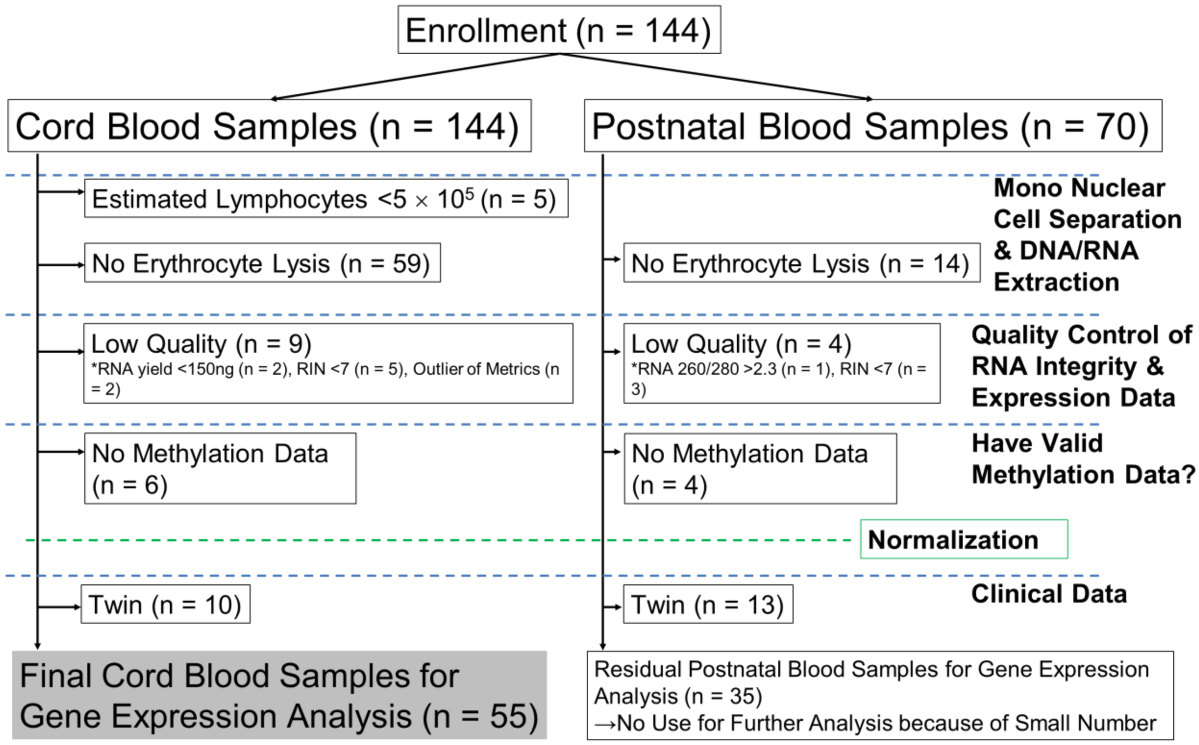

Supplementary Figure 2. Flow of RNA sample filtering.

For cord blood expression microarray analysis, from the obtained 144 samples, we excluded samples with low estimated lymphocytes $<5 \times 10^5$ (n = 5), no erythrocyte lysing buffer use in mononuclear cell isolation (n = 59), of low quality (n = 9), and samples that had no valid DNA methylation data (n = 6), and additionally, we eliminated samples of twin (n = 10) after microarray data normalization finished. The final analytic number of cord blood RNA samples was 55. And at last, for postnatal peripheral blood expression analysis, from the 70 obtained samples, we excluded samples of no erythrocyte lysing buffer use in mononuclear cell isolation (n=14), of low quality (n=4), and samples that have no valid DNA methylation data (n=4). In addition, we eliminated samples of twin (n = 13) after microarray data normalization finished. Because the residual number of postnatal peripheral blood RNA samples was so small, these samples were not used for further analysis after the normalization.

Supplementary Figure 3

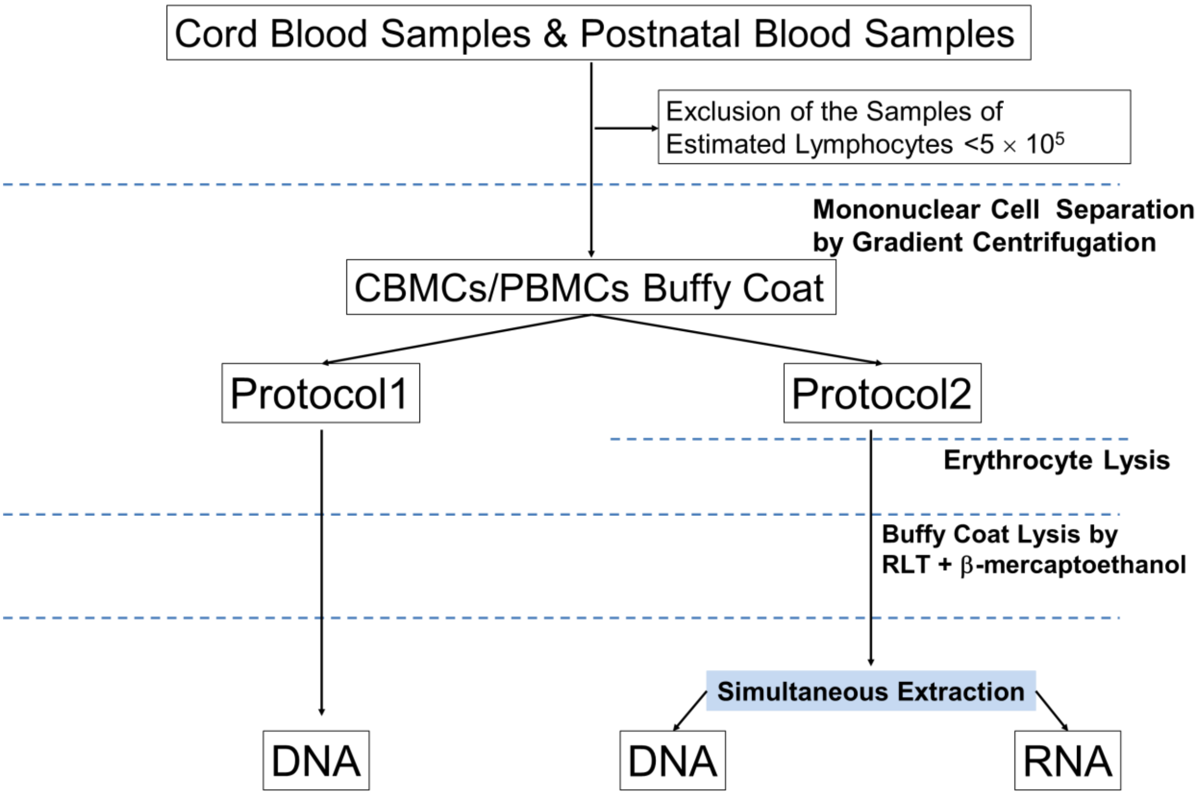

Supplementary Figure 3. Mononuclear cell separation and DNA/RNA extraction. From the obtained blood samples, cord blood mononuclear cells (CBMCs) and peripheral blood mononuclear cells (PBMCs) were separated by gradient centrifugation using Ficoll-Hypaque. When the volume of a blood sample was <1 ml, the white blood cell count and the fraction of lymphocytes out of whole white blood cells were measured by automated hematology analyzer. The samples of estimated lymphocytes $<5 \times 10^5$ were excluded. In Protocol 1, the CBMCs/PBMCs buffy coat was directly lysed with RLT buffer containing β-mercaptoethanol, and genomic DNA was extracted from the lysate of mononuclear cell buffy coat. In order to get both DNA and RNA, we had used Protocol 2 since May 2015. In Protocol2, at first the CBMCs/PBMCs buffy coat was placed into erythrocyte lysis solution. Second, the lysis solution was centrifuged, and the lysis solution containing erythrocytes was removed from the pellet. Third, the pellet was lysed with RLT buffer containing β-mercaptoethanol. Fourth, genomic DNA and total RNA were simultaneously extracted from the lysate of the pellet using the same kit in Protocol 1.

Supplementary Figure 4

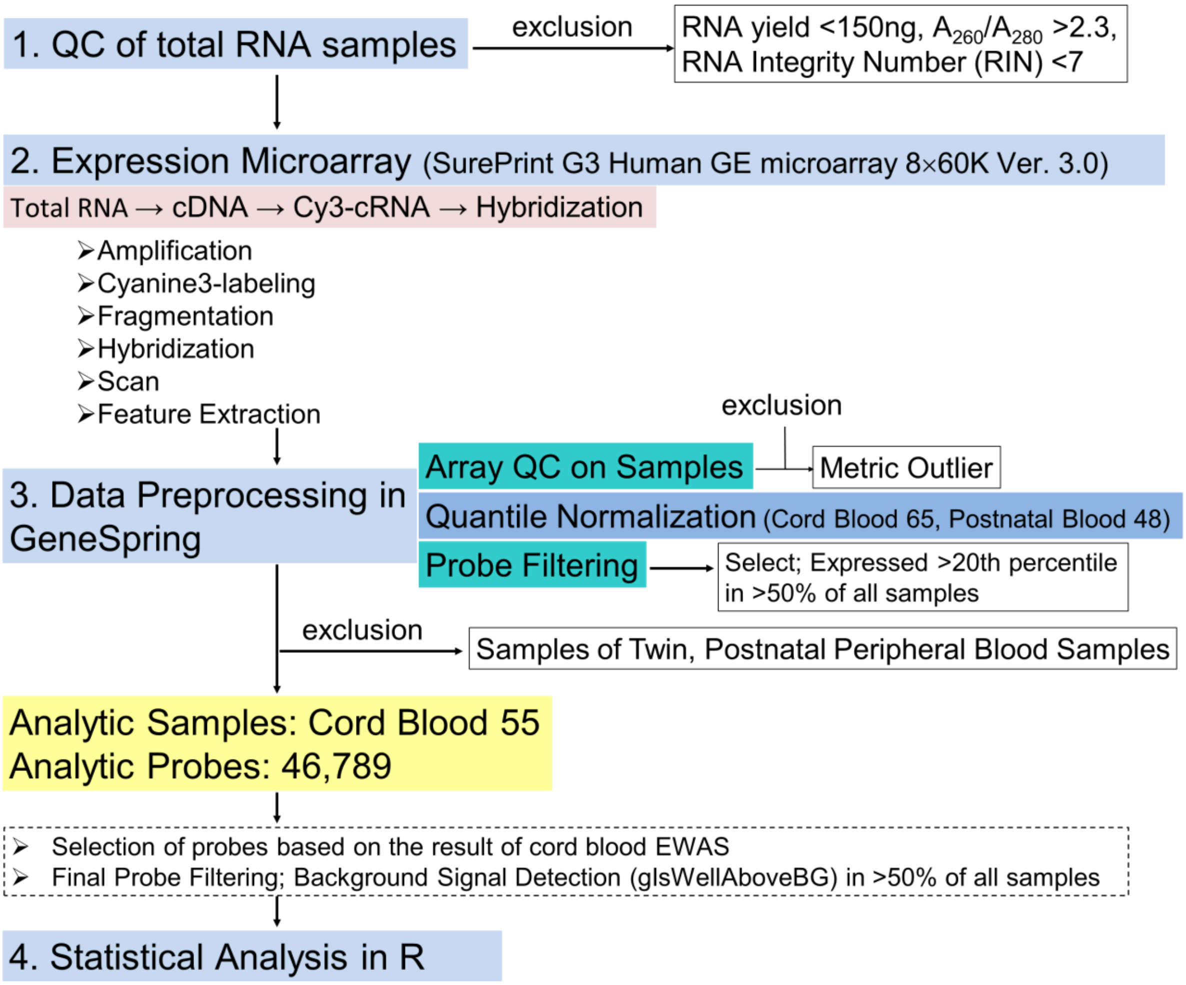

Supplementary Figure 4. Gene expression microarray analysis and data preprocessing.

Following extraction, total RNA was at first quantified and qualified using NanoDrop-1000 spectrometry (Thermo Scientific). Samples with RNA yield <150ng and with RNA absorbance ratio (A_{260}/A_{280}) >2.3 were excluded. Total RNA quality was confirmed using automated capillary electrophoresis on Agilent 2100 Bioanalyzer system (Agilent Technologies). Samples with RNA integrity number (RIN) <7 were excluded. Thereafter, 100 ng of total RNA was used to produce Cyanine 3-labeled cRNA using Low Input Quick Amp Labeling Kit according to the manufacturer's protocol (Agilent). After labeling, 600 ng of cRNA was fragmented using Agilent Gene Expression Hybridization Kit (Agilent). The fragmented cRNA was hybridized to the SurePrint G3 Human GE microarray 8×60K Ver. 3.0 (Agilent Technologies). After hybridization, the array slides were washed using Gene Expression Wash Buffer 1 and 2 (Agilent Technologies), and then scanned with the Agilent DNA Microarray Scanner (Agilent Technologies). The raw

intensity data were obtained using Agilent Feature Extraction (FE) software. Array QC on each sample, probe filtering, and normalization were performed, and samples with metrics outlier were excluded. The data set passing sample QC consisted of 65 cord blood samples and 48 postnatal peripheral blood samples. The data set was quantile normalized, and minimum expression level of all transcription among all samples was set to 1. Probes above 20th percentile expression at least 50% out of all RNA samples were selected, and furthermore, probes on the X and Y chromosomes were removed. 46,789 probes were left for further analysis. After all these preprocessing, the samples of twin (10 cord blood and 13 postnatal blood samples) were finally excluded. RNA samples of postnatal blood were small and not used for further analysis in this article. Because transcription analysis was to be done after EWAS, final probe selection and filtering was performed before transcription analysis. That is, probes were selected based on the results of cord blood EWAS, and probes with background signal detection (gIsWellAboveBG) in >50% out of all residual samples were selected. The normalized and filtered data were imported into R environment for statistical analysis.
**EWAS*: epigenome-wide association study

Supplementary Figure 5

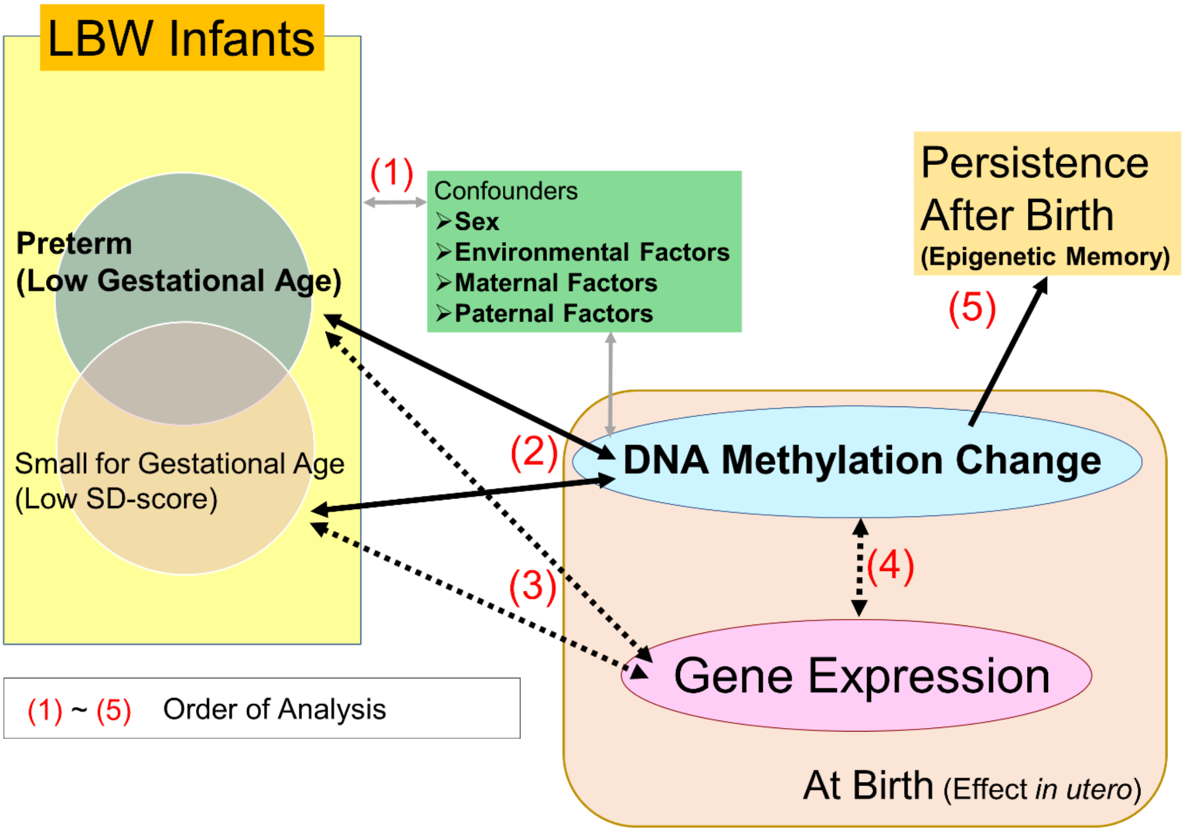

Supplementary Figure 5. Overall Analysis Framework.

*LBW infants: Low Birth Weight

Supplementary Figure 6

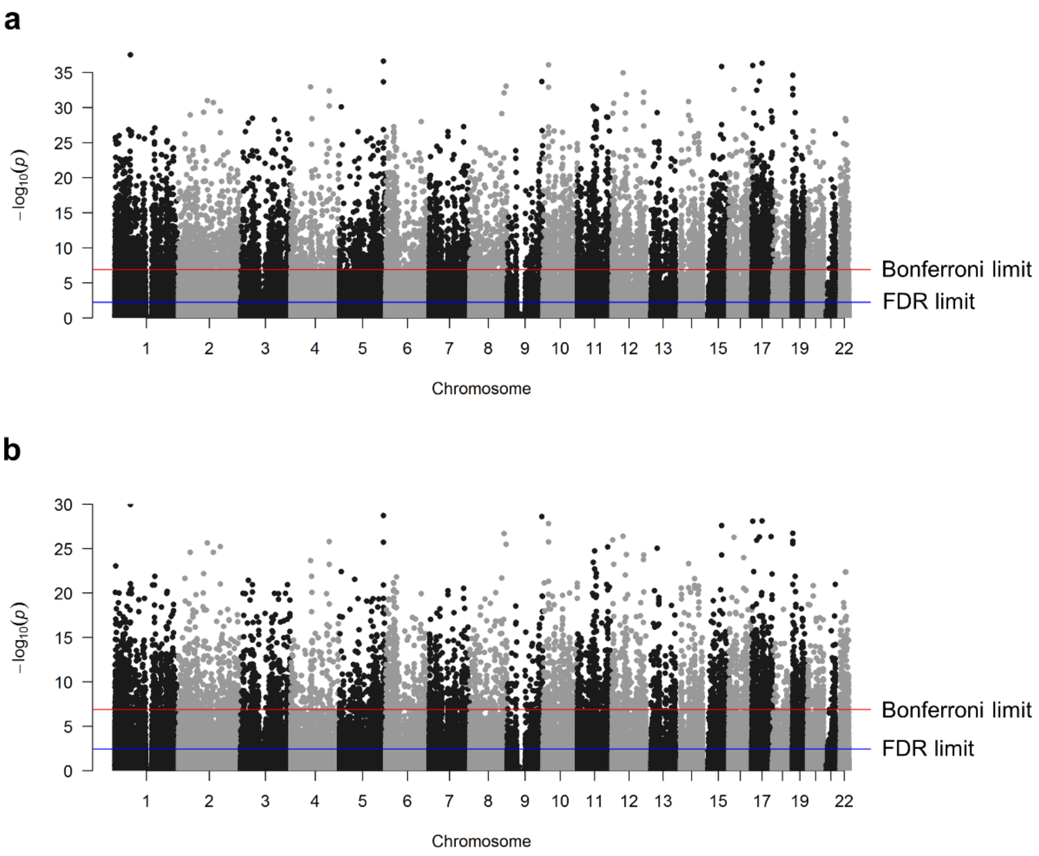

Supplementary Figure 6. **Manhattan Plot of *p*-values for associations between DNA methylation and gestational age** (n = 110, cord blood sample)**.**

a. Analysis was done by linear regression analysis of "Model 1". Association *p*-values are represented in genomic order by chromosome and position on the x-axis and $-\log_{10}p$-value on the y-axis. Horizontal red line indicates Bonferroni threshold at 0.05 and horizontal blue line indicates false discovery rate (FDR) threshold at 0.05. There were 9,823 Bonferroni-significant CpGs, and 43,930 FDR-significant CpGs. **b**. Analysis was done by linear regression analysis of "Model 2". There were 5,820 Bonferroni-significant CpGs, and 29,071 FDR-significant CpGs.

*Model 1) *objective variable*: % methylation, *predictors*: GA, birth weight SD score, *adjusted for*: infant sex, batch, cell proportion

**Model 2) *objective variable*: % methylation, *predictors*: GA, birth weight SD score, *adjusted for*: infant sex, batch, cell proportion, chorioamnionitis, idiopathic premature rupture of the membrane, preeclampsia, maternal smoking before pregnancy, maternal pre-pregnancy BMI, cesarean section

***GA*: gestational age

Supplementary Figure 7

a

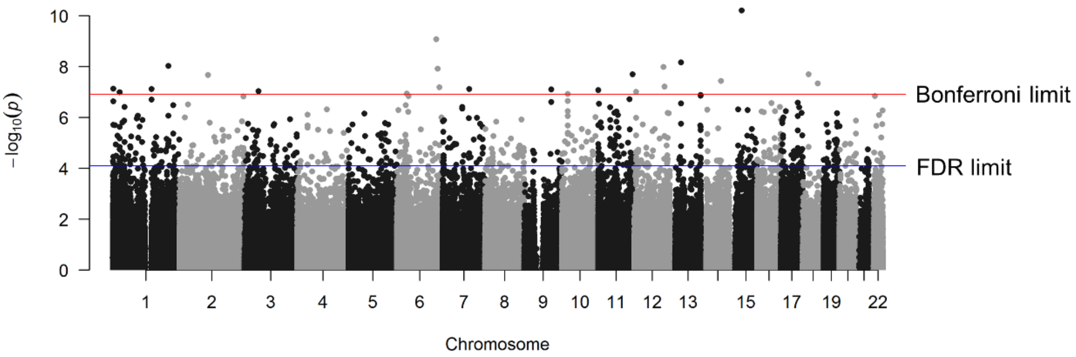

b

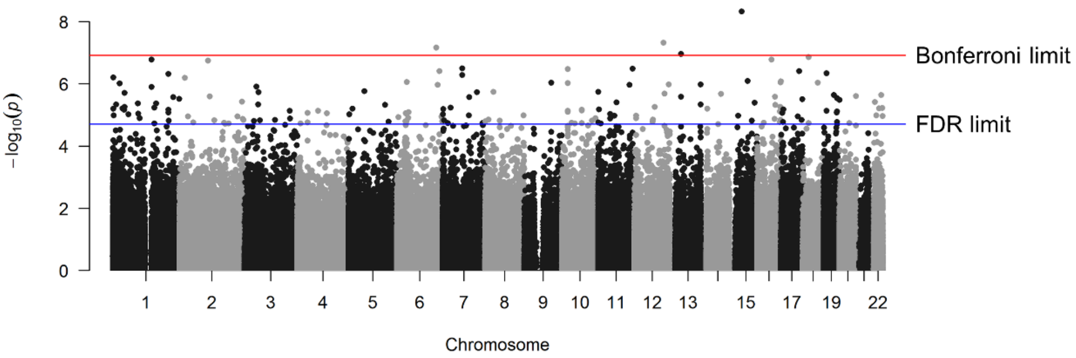

Supplementary Figure 7. **Manhattan Plot of *p*-values for associations between DNA methylation and birthweight SD score** (n = 110, cord blood sample)**.**

a. Analysis was done by linear regression analysis of "Model 1". Association *p*-values are represented in genomic order by chromosome and position on the x-axis and $-\log_{10}p$-value on the y-axis. Horizontal red line indicates Bonferroni threshold at 0.05 and horizontal blue line indicates false discovery rate (FDR) threshold at 0.05. There were 23 Bonferroni-significant CpGs, and 658 FDR-significant CpGs. **b**. Analysis was done by linear regression analysis of "Model 2". There were 4 Bonferroni-significant CpGs, and 163 FDR-significant CpGs.

*Model 1) *objective variable*: % methylation, *predictors*: GA, birth weight SD score, *adjusted for*: infant sex, batch, cell proportion

**Model 2) *objective variable*: % methylation, *predictors*: GA, birth weight SD score, *adjusted for*: infant sex, batch, cell proportion, chorioamnionitis, idiopathic premature rupture of the membrane, preeclampsia, maternal smoking before pregnancy, maternal pre-pregnancy BMI, cesarean section

****GA*: gestational age

Supplementary Figure 8

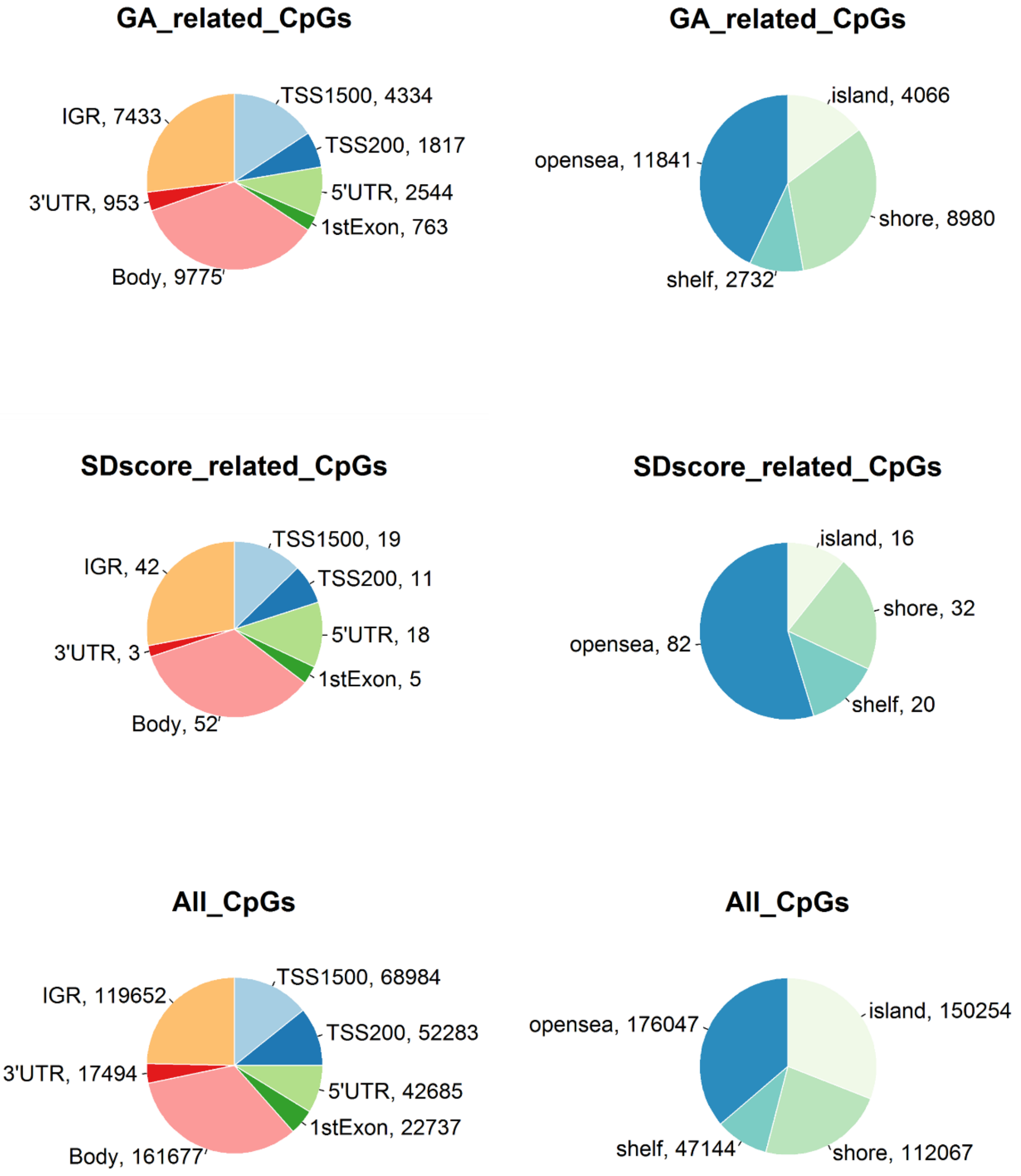

Supplementary Figure 8. Distribution of CpGs associated with gestational age and/or birth weight SD scores. Genomic distribution (left panel) and CpG content distribution (right panel) of GA-related CpGs (top panel), SD score-related CpGs (middle panel) and all CpGs contained in HumanMethylation450 BeadChip (bottom panel). These distributions are based on the result of cord blood epigenome-wide association study.
**GA*: gestational age, *SD score*: birth weight SD score

Supplementary Figure 9

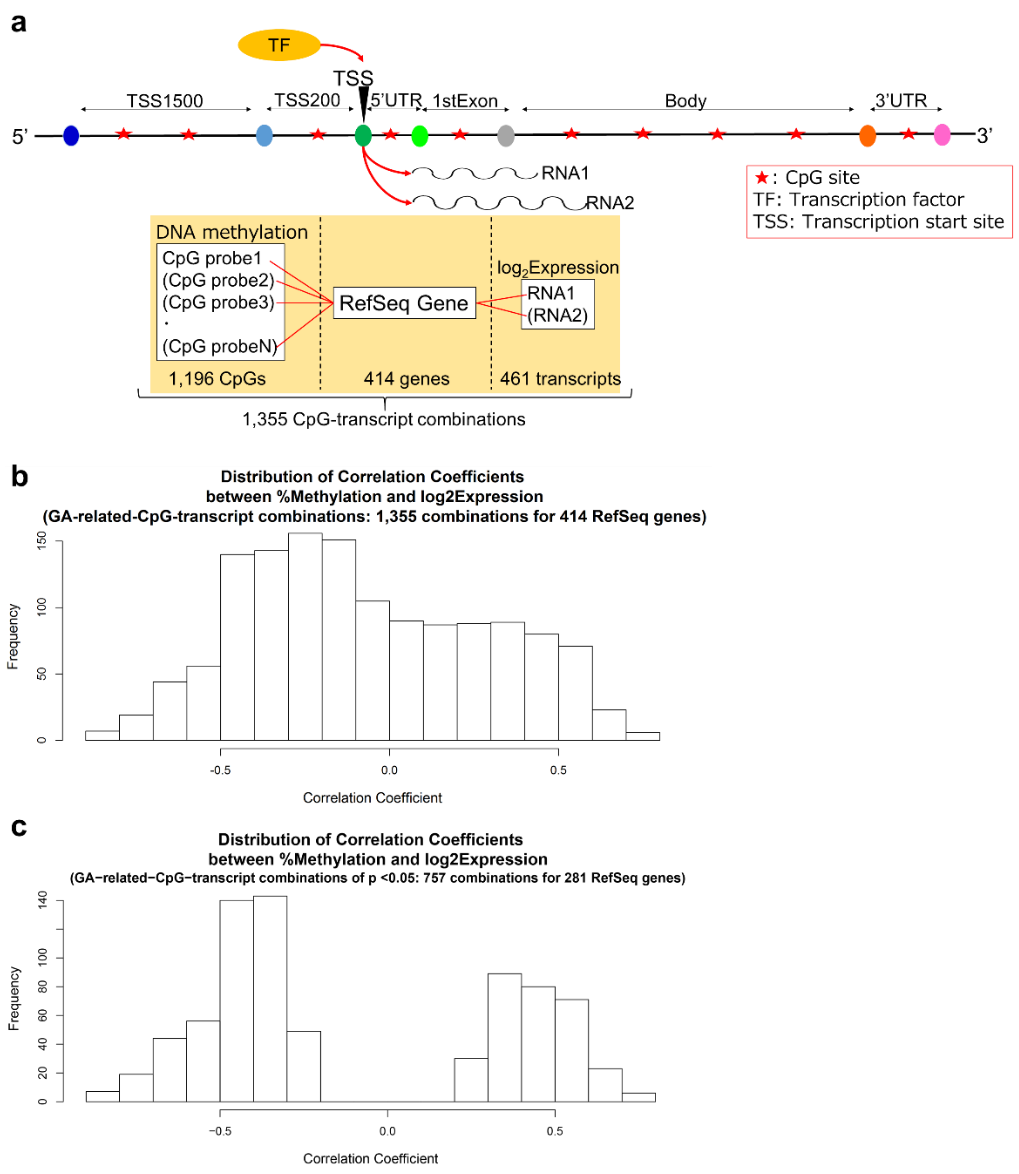

Supplementary Figure 9. Association between GA-related transcription and corresponding GA-related CpG methylation (n = 55, cord blood samples)**.**

a. 1,355 GA-related CpG-transcript pairs for interrogating direct methylation-expression relationships were generated by combining 1,196 GA-related CpGs and 461 corresponding GA-related transcripts via 414 common RefSeq genes. **b**. Histogram showing distribution of correlation coefficients between methylation and log_2-transformed expression of GA-related CpG-transcript pairs. **c**. Histogram showing distribution of correlation coefficients between methylation and log_2-transformed expression of GA-related CpG-transcript combinations, excluding the pairs of p <0.05.
**GA*: gestational age

Supplementary Figure 10

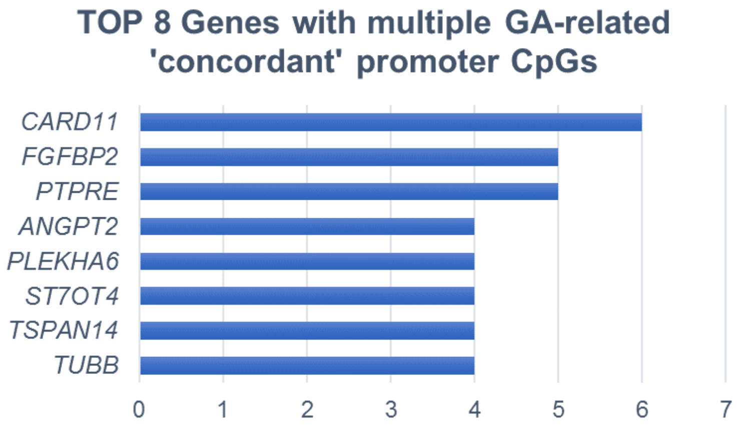

Supplementary Figure 10. Top 8 genes that have multiple GA-related CpGs of 'concordant' methylation expression relation.

Here, "Promoter Region" including TSS1500, TSS200, 5'UTR, 1st Exon. We defined negative correlation between methylation and log2-transformed gene expression as 'concordant' among the 674 expression-correlated GA-related CpGs (see Figure 4). There were 165 'concordant' promoter CpGs among the 674 CpGs. This chart means Top 8 genes that have multiple GA-related CpGs of 'concordant' methylation expression relation among the 165 'concordant' promoter CpGs.

Supplementary Figure 11

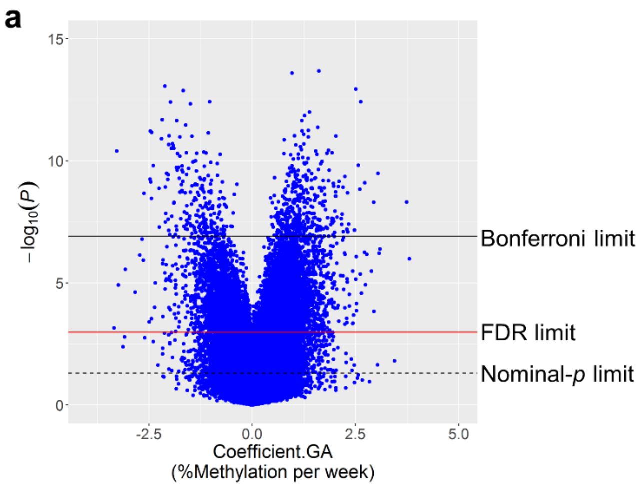

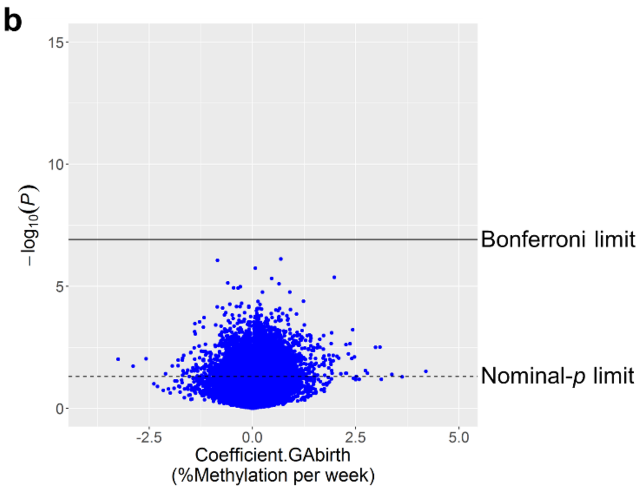

Supplementary Figure 11. Association of gestational age with DNA methylation at birth and on a postnatal day around the expected due date (n = 47, cord blood (top) and postnatal peripheral blood sample (bottom))**.**

a. Volcano plot indicating regression coefficients (x-axis) versus *p*-values (-$\log_{10}$scale) of CpG probes associated with GA at birth. All values were generated in "Model1" analysis. Top line means Bonferroni-criteria. Middle red line indicates FDR-criteria. Bottom dashed line means nominal-*p* of 0.05. **b**. Volcano plot indicating regression coefficients (x-axis) versus *p*-values (-$\log_{10}$scale) of CpG probes associated with GA at birth. All values were generated in "Model 1" analysis. Top line means Bonferroni-criteria. Bottom dashed line means nominal-*p* of 0.05.

*Model 1) *objective variable*: % Methylation, *predictors*: GA or "GA at birth", SD score or "SD score at birth", *adjusted for*: infant sex, batch, cell proportion

***GA*: gestational age, *SD score*: birth weight SD score

Supplementary Figure 12

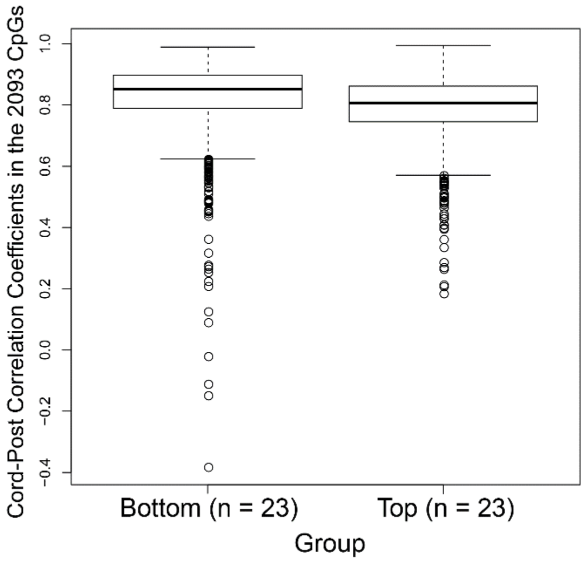

Supplementary Figure 12. Distribution of cord blood and postnatal sample (cord-post) correlation coefficients of two groups selected according to the time interval between two blood draws for 2093 candidate CpGs for GA-involved epigenetic memory.

Correlation coefficients between cord-post methylation levels among 47 infants were calculated among the 27,619 GA-related CpGs. Then, 2093 CpGs whose correlation coefficients ≥ 0.7 were selected as candidate CpGs whose methylation alteration persist after birth. Next, we selected the bottom 23 samples and top 23 samples according to the time interval between the two blood draws, and we compared the distribution of cord-post sample correlation coefficients of two groups in the 2093 candidate CpGs.

The left boxplot means the cord-post correlation coefficients' distribution of bottom 23 samples, and the right boxplot means that of top 23 samples.

Supplementary Figure 13

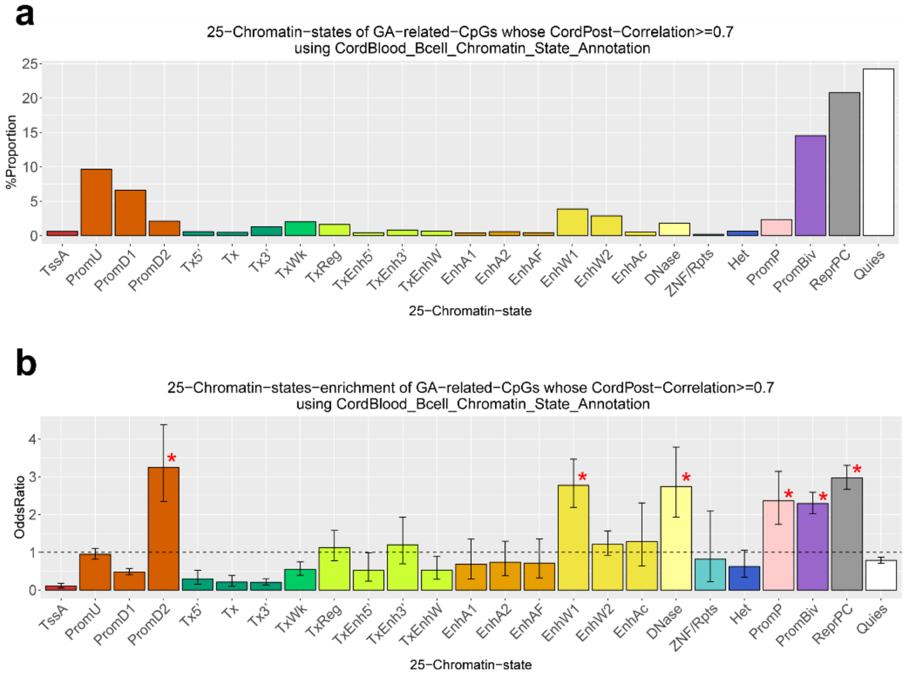

Supplementary Figure 13. Chromatin states evaluation among candidate CpGs for GA-involved epigenetic memory by using cord blood B cell-based annotation in ChromHMM (n = 47, cord blood samples and postnatal blood samples)**.**

a. Distribution of the 25 Chromatin states for GA-related CpGs whose cord post correlation of % methylation ≥ 0.7 using cord blood B cell-based annotation in ChromHMM. **b**. Enrichment of the 25 Chromatin states for GA-related CpGs whose cord post correlation of % methylation ≥ 0.7 using cord blood B cell-based annotation in ChromHMM. Error bars mean 95%CI (confidence interval). * denotes the enriched chromatin state which was significant at Bonferroni-criteria (0.05/25) and of odds ratio ≥ 1 (black dashed line).

**GA*: gestational age, *EWAS*: epigenome-wide association study

Following Abbreviations were defined in ChromHMM; TssA: Active TSS, PromU: Promoter upstream TSS, PromD1: Promoter downstream with DNase, PromD2: Promoter downstream TSS, Tx5': Transcription 5', Tx: Transcription, Tx3': Transcription 3', TxWk: Weak Transcription, TxReg: Transcription Regulatory, TxEnh5': Transcription 5' Enhancer, TxEnh3': Transcription 3' Enhancer, TxEnhW: Transcription Weak Enhancer, EnhA1: Active Enhancer 1, EnhA2: Active Enhancer 2, EnhAF: Active Enhancer Flank, EnhW1: Weak Enhancer 1, EnhW2: Weak Enhancer 2, EnhAc: Enhancer Acetylation Only, DNase: DNase only, ZNF/Rpts: ZNF genes & repeats, Het: Heterochromatin, PromP: Poised Promoter, PromBiv: Bivalent Promoter, ReprPC: Repressed Polycomb, Qies: Quiescent/Low.

Supplementary Figure 14

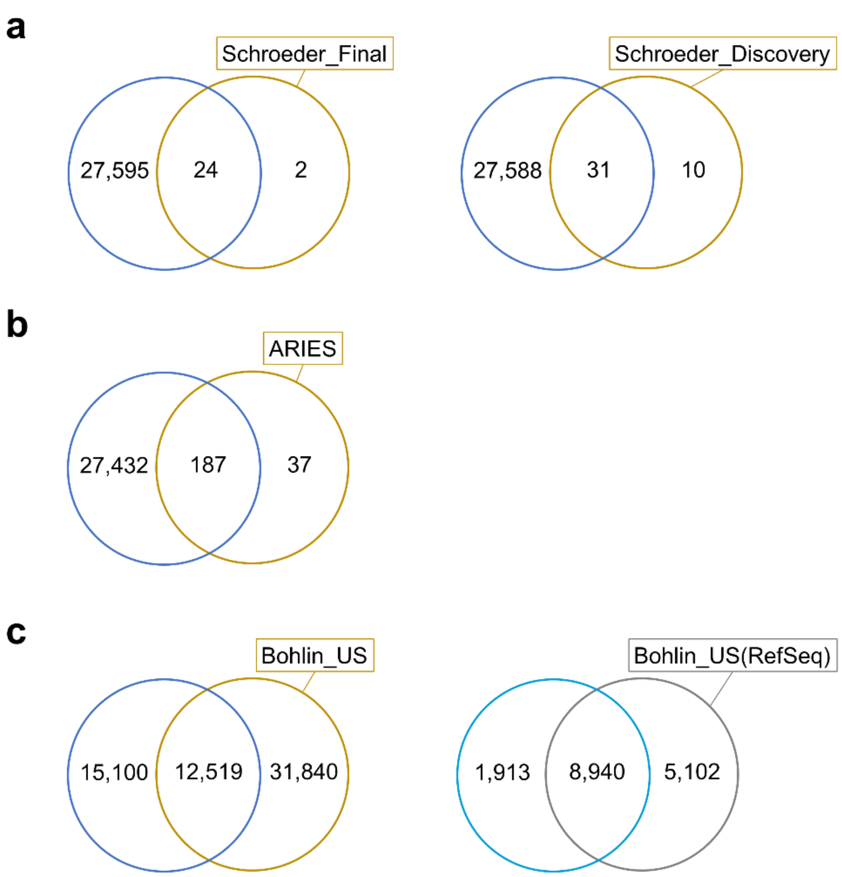

Supplementary Figure 14. Comparison with the GA-related CpGs identified in previous EWAS.

In each panel except for one, left circles with blue line mean the GA-related CpGs identified in the current study. In each panel except for one, intersect in Venn diagram indicates the CpGs that was found in both groups in the same direction of GA's effect on methylation.

a Comparison with Schroeder *et al.* study[6]. Left panel indicates the comparison with GA-related CpGs that was determined by both discovery cohort and replication cohort, and right panel means the comparison with CpGs decided only by discovery cohort. In each panel, right circle is the GA-related CpGs in Schroeder *et al.* study. **b** Comparison with Simpkin *et al.* ARIES cohort study[7]. Right circle is the GA-related CpGs in ARIES' study. **c** Comparison with Bohlin *et al.* study dealing with MoBa cohort data[8]. In left panel, right circle is the CpGs associated with the gestational age determined by ultrasonography at the threshold of FDR <0.05. Right panel means the comparison of corresponding RefSeq genes which is nearest to GA-related CpGs within 250bp upstream or downstream. In this panel, intersect does not contain any meaning of directionality.

Supplementary Figure 15

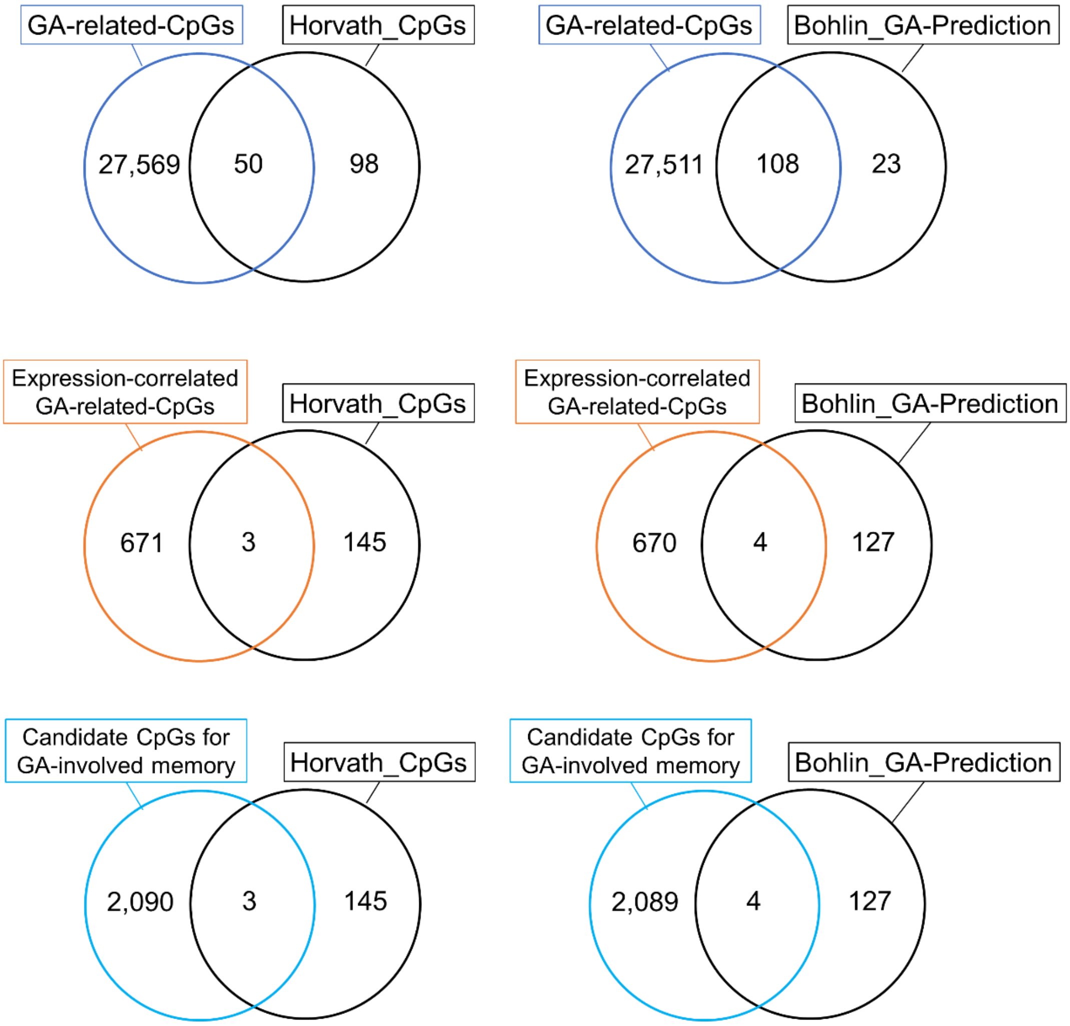

Supplementary Figure 15. Comparison with the CpGs which were selected for the purpose of predicting gestational age utilizing their methylation data.

In each panel, intersect in Venn diagram does not contain any meaning of effect's direction. Top panel means the comparison of GA-prediction CpGs (right circle) and our GA-related CpGs (left circle). Middle panel means the comparison of GA-prediction CpGs (right circle) and GA-related CpGs which were significantly correlated with corresponding transcriptions in the present study (left circle). Bottom panel means the comparison of GA-prediction CpGs (right circle) and GA-related CpGs of correlation coefficient ≥ 0.7 between cord blood and postnatal blood methylation in this study (left circle). In left panel, GA-prediction CpGs were Horvath's CpGs investigated by Knight *et al*[9]. In right panel, GA-prediction CpGs were the CpGs determined by Bohlin *et al.* utilizing MoBa cohort data[8].

Supplementary Table 1. Pregnancy- and delivery-related characteristics of 110 mother-infant pairs.

Prenatal Variable	Mean (SD)	Median	N (%)
Paternal age	36.4 (6.7)	35.5	
<25 years			2 (1.8)
25 ~ 30 years			11 (10.0)
30 ~ 35 years			31 (28.2)
35 ~ 40 years			33 (30.0)
>40 years			33 (30.0)
Paternal BMI	23.1 (2.9)	22.7	
<18.5 kg/m^2			2 (1.8)
18.5 ~ 25 kg/m^2			82 (74.5)
25 ~ 30 kg/m^2			23 (20.9)
>30 kg/m^2			3 (2.7)

*Since the table containing all the values exceeds one page, other descriptive characteristics that cannot be written in Table 1 are shown here.

Supplementary Table 2. **Association of prenatal covariates with gestational age – Result of univariate linear regression analysis** (n = 110). Regression coefficients (Est), 95% confidence intervals (CI) and *p*-values are reported as a week's change in gestational age for two standard deviations increases in continuous prenatal variables, or for comparing the two categories of binary prenatal variables.

	Est	95%CI	*p*-value
Birth weight SD score	2.6	(0.83, 4.4)	0.0046
Male	-2.0	(-3.9, -0.21)	0.029
Parity	-0.41	(-2.3, 1.5)	0.67
Cesarean section	-3.1	(-5.4, -0.86)	0.0075
Maternal age	0.90	(-0.96, 2.8)	0.34
Maternal pre-pregnancy BMI	-2.5	(-4.3, -0.70)	0.0071
Paternal age	-0.53	(-2.4, 1.3)	0.58
Paternal BMI	-0.57	(-2.4, 1.3)	0.54
Assisted reproductive technology (ART)	0.40	(-1.8, 2.6)	0.72
Maternal smoking before pregnancy	-6.1	(-9.7, -2.5)	0.0011
Gestational diabetes mellitus (GDM)	-0.24	(-5.2, 4.7)	0.93
Chorioamnionitis (CAM)	-4.7	(-6.7, -2.7)	1.1E-05
Idiopathic premature rupture of the membrane (iPROM)	-2.9	(-6.0, 0.12)	0.060
Preeclampsia	-1.6	(-4.0, 0.80)	0.19
Placenta previa	-0.0057	(-3.2, 3.2)	0.99

Supplementary Table 3. **Association of prenatal covariates with birth weight SD scores – Result of univariate linear regression analysis** (n = 110)**.** Regression coefficients (Est), 95% confidence intervals (CI) and *p*-values are reported as change in birth weight SD scores for two standard deviations increases in continuous prenatal variables, or for comparing the two categories of binary prenatal variables.

	Est	95%CI	*p*-value
Gestational age	0.76	(0.24, 1.3)	0.0046
Male	0.058	(-0.48, 0.60)	0.83
Parity	-0.46	(-1.0, 0.080)	0.094
Cesarean section	-0.59	(-1.3, 0.087)	0.087
Maternal age	0.23	(-0.31, 0.77)	0.40
Maternal pre-pregnancy BMI	0.24	(-0.29, 0.78)	0.37
Paternal age	-0.031	(-0.57, 0.51)	0.91
Paternal BMI	0.25	(-0.29, 0.79)	0.36
Assisted reproductive technology (ART)	0.18	(-0.46, 0.82)	0.58
Maternal smoking before pregnancy	0.79	(-0.31, 1.9)	0.16
Gestational diabetes mellitus (GDM)	1.1	(-0.35, 2.5)	0.14
Chorioamnionitis (CAM)	0.19	(-0.45, 0.83)	0.56
Idiopathic premature rupture of the membrane (iPROM)	0.51	(-0.38, 1.4)	0.26
Preeclampsia	-1.1	(-1.8, -0.44)	0.0014
Placenta previa	-0.39	(-1.3, 0.55)	0.41

Supplementary Table 4. **Association of prenatal covariates with gestational age – Result of multivariate linear regression analysis** (n = 110)**.** Multivariate linear regression analysis was performed to reconfirm the relation with gestational age, adjusted for each other except for the objective variable. Regression coefficients (Est), 95% confidence intervals (CI) and *p*-values are reported as a week's change in gestational age for two standard deviations increases in continuous prenatal variables, or for comparing the two categories of binary prenatal variables.

	Est	95%CI	*p*-value
Birth weight SD score	2.6	(1.1, 4.1)	0.0011
Male	-1.1	(-2.6, 0.41)	0.15
Cesarean section	-2.5	(-4.4, -0.68)	0.0081
Maternal pre-pregnancy BMI	-0.68	(-2.3, 0.97)	0.42
Maternal smoking before pregnancy	-4.4	(-7.7, -1.2)	0.0076
Chorioamnionitis (CAM)	-4.6	(-6.5, -2.7)	6.8E-06
Idiopathic premature rupture of the membrane (iPROM)	-4.7	(-7.2, -2.3)	0.00027
Preeclampsia	-2.6	(-4.7, -0.49)	0.016

Supplementary Table 5. Association of prenatal covariates with birth weight SD scores – Result of multivariate linear regression analysis (n = 110)**.** Multivariate linear regression analysis was performed to reconfirm the relation with birth weight SD score, adjusted for each other except for the objective variable. Regression coefficients (Est), 95% confidence intervals (CI) and *p*-values are reported as a week's change in gestational age for two standard deviations increases in continuous prenatal variables, or for comparing the two categories of binary prenatal variables.

	Est	95%CI	*p*-value
Gestational age	1.1	(0.44, 1.7)	0.0011
Male	-0.10	(-0.64, 0.43)	0.70
Cesarean section	-0.29	(-0.95, 0.38)	0.40
Maternal pre-pregnancy BMI	0.50	(-0.062, 1.1)	0.080
Maternal smoking before pregnancy	0.78	(-0.37, 1.9)	0.18
Chorioamnionitis (CAM)	0.40	(-0.33, 1.1)	0.28
Idiopathic premature rupture of the membrane (iPROM)	0.87	(-0.036, 1.8)	0.060
Preeclampsia	-0.87	(-1.6, -0.14)	0.020

Supplementary Table 8. **Confirmation of the results of 27,619 GA-related CpGs based on "Model 1" and "Model 2" − Comparison with the results of sensitivity analysis by using Model 1 adjusted for each of the 6 prenatal covariates of "Model 2".**

<table>
<tr><th colspan="4">Number of CpGs associated with GA (significant level: BH-FDR < 0.05; 410735 tests)</th></tr>
<tr><th rowspan="2">"GA-related CpGs" (common with Model1 and Model2)</th><th colspan="2">common with Model1, Model2, and each sensitivity analysis</th><th rowspan="2">common with Model1, Model2, and all the sensitivity analyses</th></tr>
<tr><th>Covariate added to the covariates of Model1</th><th>Number of CpGs</th></tr>
<tr><td rowspan="6">27619</td><td>Delivery mode</td><td>27199</td><td rowspan="6">26202</td></tr>
<tr><td>Maternal pre-pregnancy BMI</td><td>27218</td></tr>
<tr><td>Smoking before pregnancy</td><td>27130</td></tr>
<tr><td>Chorioamnionitis</td><td>27068</td></tr>
<tr><td>Idiopathic PROM</td><td>27331</td></tr>
<tr><td>Preeclampsia</td><td>27519</td></tr>
</table>

Supplementary Table 9. Confirmation of the results of 150 SD score-related CpGs based on “Model 1” and “Model 2” − Comparison with the results of sensitivity analysis by using Model 1 adjusted for each of the 6 prenatal covariates of “Model 2”.

Number of CpGs associated with SD scores (significant level: BH-FDR < 0.05; 410735 tests)			
"SD score-related CpGs" (common with Model1 and Model2)	common with Model1, Model2, and each sensitivity analysis		common with Model1, Model2, and all the sensitivity analyses
	Covariate added to the covariates of Model1	Number of CpGs	
150	Delivery mode	149	141
	Maternal pre-pregnancy BMI	149	
	Smoking before pregnancy	142	
	Chorioamnionitis	150	
	Idiopathic PROM	147	
	Preeclampsia	150	

Supplementary Table 10. **Enrichment in KEGG pathway for the GA-related CpGs.** For 27,619 GA-related CpGs identified in cord blood epigenome-wide association study, only the gene entries which had at least 2 CpGs on its promoter region were used for enrichment analysis in KEGG pathway.

*ER-*p*-value: enrichment *p*-value generated in DAVID enrichment analysis.

**ER-FDR: adjusted enrichment *p*-value using Benjamini-Hochberg procedure.

****GA*: gestational age

****: ER-FDR > 0.1, suggestive

[Positively GA-related CpGs]

KEGG pathway	Count	ER-*p*-Value*	ER-FDR**
ECM-receptor interaction	16	9.8E-05	0.025
Adrenergic signaling in cardiomyocytes	21	2.1E-04	0.026
Tight junction	19	7.0E-04	0.044
PI3K-Akt signaling pathway	36	5.3E-04	0.045
Cell adhesion molecules (CAMs)	19	1.1E-03	0.054
Rap1 signaling pathway	23	3.9E-03	0.095
Gastric acid secretion	12	2.6E-03	0.10
Estrogen signaling pathway	14	3.8E-03	0.10
Ras signaling pathway	24	4.6E-03	0.10
Focal adhesion	23	3.1E-03	0.11****
Oxytocin signaling pathway	19	3.6E-03	0.11****
Signaling pathways regulating pluripotency of stem cells	17	5.7E-03	0.12****

[Negatively GA-related CpGs]

KEGG pathway	Count	ER-*p*-Value*	ER-FDR**
Inflammatory bowel disease (IBD)	14	2.1E-07	5.0E-05
Rheumatoid arthritis	15	1.7E-06	2.0E-04
Influenza A	19	3.0E-05	1.8E-03
Cytokine-cytokine receptor interaction	22	4.4E-05	2.1E-03
Intestinal immune network for IgA production	10	2.7E-05	2.2E-03
Viral myocarditis	10	1.3E-04	4.5E-03
Tuberculosis	18	1.3E-04	5.1E-03
NF-kappa B signaling pathway	12	1.9E-04	5.7E-03

Allograft rejection	8	2.4E-04	6.3E-03
Staphylococcus aureus infection	9	4.9E-04	0.012
HTLV-I infection	21	5.5E-04	0.012
Herpes simplex infection	17	5.9E-04	0.012
Graft-versus-host disease	7	8.3E-04	0.015
Primary immunodeficiency	7	9.8E-04	0.017
Chemokine signaling pathway	16	2.0E-03	0.029
Measles	13	2.2E-03	0.030
Autoimmune thyroid disease	8	2.0E-03	0.031
Type I diabetes mellitus	7	3.0E-03	0.036
T cell receptor signaling pathway	11	2.9E-03	0.038
Leishmaniasis	9	3.0E-03	0.038
Asthma	6	3.4E-03	0.038
Jak-STAT signaling pathway	13	4.4E-03	0.047
Malaria	7	6.6E-03	0.067
Toxoplasmosis	11	7.7E-03	0.075
Natural killer cell mediated cytotoxicity	11	9.7E-03	0.089
Cell adhesion molecules (CAMs)	12	0.010	0.091
Amoebiasis	10	0.011	0.095

Supplementary Table 13. **Enrichment in KEGG pathway for the GA-related expression probes selected based on the criteria of FDR.** The result of pathway enrichment analysis after transcription association analysis. Here, probe selection was based on a threshold of FDR ≤ 0.05 in the transcription association analysis on gestational age. Tables in this section show KEGG pathway categories with enrichment *p*-value ≤ 0.1.

*ER-*p*-value: enrichment *p*-value generated in DAVID enrichment analysis.

**ER-FDR: adjusted enrichment *p*-value using Benjamini-Hochberg procedure.

****GA*: gestational age

[Negatively GA-related expression probes]

KEGG pathway	Count	ER-*p*-Value*	ER-FDR**
Platelet activation	11	1.9E-06	3.0E-04
PI3K-Akt signaling pathway	13	4.9E-04	0.039
Focal adhesion	10	5.3E-04	0.028
Rap1 signaling pathway	9	2.6E-03	0.10
ECM-receptor interaction	6	3.1E-03	0.094
Proteoglycans in cancer	8	7.6E-03	0.19
Cell cycle	6	0.013	0.27
Pathways in cancer	11	0.014	0.24
MicroRNAs in cancer	8	0.044	0.55
Ras signaling pathway	7	0.044	0.52
Hematopoietic cell lineage	4	0.073	0.67
Gap junction	4	0.079	0.67
Prostate cancer	4	0.079	0.67

[Positively GA-related expression probes]

KEGG pathway	Count	ER-*p*-Value*	ER-FDR**
B cell receptor signaling pathway	4	0.023	0.97
Epstein-Barr virus infection	6	0.026	0.87
Osteoclast differentiation	5	0.028	0.77
Graft-versus-host disease	3	0.034	0.74
MicroRNAs in cancer	7	0.039	0.71
Allograft rejection	3	0.042	0.67
Phagosome	5	0.046	0.65

Type I diabetes mellitus	3	0.053	0.65
cGMP-PKG signaling pathway	5	0.058	0.65
T cell receptor signaling pathway	4	0.062	0.63
Tuberculosis	5	0.070	0.64
HTLV-I infection	6	0.075	0.63
Autoimmune thyroid disease	3	0.077	0.62
Herpes simplex infection	5	0.077	0.59
Toxoplasmosis	4	0.086	0.61
Sphingolipid signaling pathway	4	0.089	0.60
Viral myocarditis	3	0.090	0.58
Natural killer cell mediated cytotoxicity	4	0.093	0.57

Supplementary Table 14. Enrichment in KEGG pathway for the GA-related expression probes selected based on the criteria of nominal *p*-value. The result of pathway enrichment analysis after transcription association analysis. Here, probe selection was based on a threshold of nominal *p*-value <0.05 in the analysis of gene expression changes associated with gestational age.

*ER-*p*-value: enrichment *p*-value generated in DAVID enrichment analysis.

**ER-FDR: adjusted enrichment *p*-value using Benjamini-Hochberg procedure.

****GA*: gestational age

****: ER-FDR > 0.1, suggestive

[Negatively GA-related expression probes]

KEGG pathway	Count	ER-*p*-Value*	ER-FDR**
Platelet activation	16	1.7E-05	3.9E-03
PI3K-Akt signaling pathway	27	4.2E-05	4.9E-03
Rap1 signaling pathway	19	1.4E-04	0.011
Cell cycle	14	1.7E-04	9.7E-03
Focal adhesion	18	3.3E-04	0.015
ECM-receptor interaction	11	4.5E-04	0.018
Proteoglycans in cancer	16	2.0E-03	0.063
Gap junction	10	2.0E-03	0.057
Arginine and proline metabolism	7	5.1E-03	0.12****
Hematopoietic cell lineage	9	5.9E-03	0.13****

[Positively GA-related expression probes]

KEGG pathway	Count	ER-*p*-Value*	ER-FDR**
Graft-versus-host disease	13	6.7E-10	1.6E-07
Allograft rejection	13	3.1E-09	3.7E-07
Type I diabetes mellitus	13	1.6E-08	1.2E-06
Viral myocarditis	14	7.5E-08	4.5E-06
Autoimmune thyroid disease	13	2.1E-07	1.0E-05
HTLV-I infection	26	4.7E-06	1.9E-04
Inflammatory bowel disease (IBD)	12	1.5E-05	5.0E-04
Cell adhesion molecules (CAMs)	17	4.6E-05	1.4E-03
NF-kappa B signaling pathway	13	5.9E-05	1.6E-03
Antigen processing and presentation	12	7.7E-05	1.8E-03

Hematopoietic cell lineage	12	2.1E-04	4.7E-03
Rheumatoid arthritis	12	2.9E-04	5.8E-03
Herpes simplex infection	18	3.0E-04	5.5E-03
Asthma	7	5.9E-04	0.010
Leishmaniasis	10	9.4E-04	0.015
Intestinal immune network for IgA production	8	1.3E-03	0.020
Epstein-Barr virus infection	16	3.5E-03	0.048
Cytokine-cytokine receptor interaction	18	3.8E-03	0.049
Tuberculosis	15	4.6E-03	0.056
Insulin resistance	11	5.4E-03	0.063
Toxoplasmosis	11	9.9E-03	0.11****
B cell receptor signaling pathway	8	0.012	0.12****
Signaling pathways regulating pluripotency of stem cells	12	0.012	0.12****
Staphylococcus aureus infection	7	0.013	0.12****
Adipocytokine signaling pathway	8	0.013	0.11****
Basal cell carcinoma	7	0.014	0.12****
TNF signaling pathway	10	0.014	0.12****
Pathways in cancer	24	0.014	0.11****

Supplementary Table 16. Distribution and enrichment of the 25 chromatin states among 'discordant' GA-related CpGs that had positive correlation between methylation and corresponding $\log_2$-transformed transcription using cord blood T cell-based annotation provided by ChromHMM.

*Bold characters mean enriched chromatin states which met the criteria of Bonferroni-significance (*p*-value <0.05/25) and odds ratio ≥ 1.

***OR*: odds ratio, *CI*: confidence interval

*****Following Abbreviations of 25 chromatin states are defined in ChromHMM**; TssA: Active TSS, PromU: Promoter upstream TSS, PromD1: Promoter downstream with DNase, PromD2: Promoter downstream TSS, Tx5': Transcription 5', Tx: Transcription, Tx3': Transcription 3', TxWk: Weak Transcription, TxReg: Transcription Regulatory, TxEnh5': Transcription 5' Enhancer, TxEnh3': Transcription 3' Enhancer, TxEnhW: Transcription Weak Enhancer, EnhA1: Active Enhancer 1, EnhA2: Active Enhancer 2, EnhAF: Active Enhancer Flank, EnhW1: Weak Enhancer 1, EnhW2: Weak Enhancer 2, EnhAc: Enhancer Acetylation Only, DNase: DNase only, ZNF/Rpts: ZNF genes & repeats, Het: Heterochromatin, PromP: Poised Promoter, PromBiv: Bivalent Promoter, ReprPC: Repressed Polycomb, Qies: Quiescent/Low.

25-Chromatin-State (T cell-based)	Count	Proportion (%)	OR	95%CI	*p*-value
Active TSS (TssA)	1	1.2	0.22	(0.0054, 1.2)	0.16
Promoter upstream TSS (PromU)	15	17.9	1.8	(0.97, 3.2)	0.048
Promoter downstream with DNase (PromD1)	5	6.0	0.54	(0.17, 1.3)	0.21
Promoter downstream TSS (PromD2)	3	3.6	3.8	(0.77, 12)	0.048
Transcription 5' (Tx5')	3	3.6	2.0	(0.40, 6.0)	0.20
Transcription (Tx)	0	0.0	0.0	(0, 2.2)	0.42
Transcription 3' (Tx3')	0	0.0	0.0	(0, 0.86)	0.023
Weak Transcription (TxWk)	1	1.2	0.32	(0.0080, 1.8)	0.38
Transcription Regulatory (TxReg)	0	0.0	0.0	(0, 3.8)	1
Transcription 5' Enhancer (TxEnh5')	0	0.0	0.0	(0, 8.3)	1
Transcription 3' Enhancer (TxEnh3')	1	1.2	1.7	(0.043, 9.9)	0.44
Transcription Weak Enhancer	3	3.6	2.0	(0.40, 6.0)	0.20

(TxEnhW)					
Active Enhancer 1 (EnhA1)	0	0.0	0.0	(0, 12)	1
Active Enhancer 2 (EnhA2)	4	4.8	7.9	(2.1, 21)	**0.0019**
Active Enhancer Flank (EnhAF)	2	2.4	4.7	(0.56, 17.5)	0.071
Weak Enhancer 1 (EnhW1)	6	7.1	3.6	(1.3, 8.3)	0.0080
Weak Enhancer 2 (EnhW2)	0	0.0	0.0	(0, 1.4)	0.12
Enhancer Acetylation Only (EnhAc)	0	0.0	0.0	(0, 88)	1
DNase only (DNase)	0	0.0	0.0	(0, 7.0)	1
ZNF genes & repeats (ZNF/Rpts)	0	0.0	0.0	(0, 25)	1
Heterochromatin (Het)	1	1.2	1.9	(0.048, 11)	0.41
Poised Promoter (PromP)	0	0.0	0.0	(0, 3.9)	1
Bivalent Promoter (PromBiv)	8	9.5	1.2	(0.52, 2.6)	0.54
Repressed Polycomb (ReprPC)	19	22.6	3.8	(2.2, 6.4)	**6.0E-06**
Quiescent/Low (Quies)	12	14.3	0.36	(0.18, 0.67)	0.00037

Supplementary Table 17. Distribution and enrichment of the 25 chromatin states among 'concordant' GA-related CpGs that had positive correlation between methylation and corresponding $\log_2$-transformed transcription using cord blood T cell-based annotation provided by ChromHMM.

*Bold characters mean enriched chromatin states which met the criteria of Bonferroni-significance (*p*-value <0.05/25) and odds ratio ≥ 1.

***OR*: odds ratio, *CI*: confidence interval

*****Following Abbreviations of 25 chromatin states are defined in ChromHMM**; TssA: Active TSS, PromU: Promoter upstream TSS, PromD1: Promoter downstream with DNase, PromD2: Promoter downstream TSS, Tx5': Transcription 5', Tx: Transcription, Tx3': Transcription 3', TxWk: Weak Transcription, TxReg: Transcription Regulatory, TxEnh5': Transcription 5' Enhancer, TxEnh3': Transcription 3' Enhancer, TxEnhW: Transcription Weak Enhancer, EnhA1: Active Enhancer 1, EnhA2: Active Enhancer 2, EnhAF: Active Enhancer Flank, EnhW1: Weak Enhancer 1, EnhW2: Weak Enhancer 2, EnhAc: Enhancer Acetylation Only, DNase: DNase only, ZNF/Rpts: ZNF genes & repeats, Het: Heterochromatin, PromP: Poised Promoter, PromBiv: Bivalent Promoter, ReprPC: Repressed Polycomb, Qies: Quiescent/Low.

25-Chromatin-State (T cell-based)	Count	Proportion (%)	OR	95%CI	*p*-value
Active TSS (TssA)	0	0.0	0.0	(0, 0.41)	3.0E-04
Promoter upstream TSS (PromU)	37	22.4	2.4	(1.6, 3.5)	**1.1E-05**
Promoter downstream with DNase (PromD1)	21	12.7	1.2	(0.74, 2.0)	0.37
Promoter downstream TSS (PromD2)	5	3.0	3.2	(1.0, 7.7)	0.022
Transcription 5' (Tx5')	0	0.0	0.0	(0, 1.2)	0.081
Transcription (Tx)	2	1.2	0.59	(0.071, 2.2)	0.78
Transcription 3' (Tx3')	0	0.0	0.0	(0, 0.43)	4.4E-04
Weak Transcription (TxWk)	2	1.2	0.33	(0.039, 1.2)	0.14
Transcription Regulatory (TxReg)	10	6.1	5.5	(2.6, 10)	**2.8E-05**
Transcription 5' Enhancer (TxEnh5')	4	2.4	4.6	(1.2, 12)	0.012
Transcription 3' Enhancer (TxEnh3')	0	0.0	0.0	(0, 3.3)	0.63
Transcription Weak Enhancer (TxEnhW)	4	2.4	1.3	(0.36, 3.5)	0.55
Active Enhancer 1 (EnhA1)	3	1.8	4.8	(0.98, 14)	0.027

Active Enhancer 2 (EnhA2)	8	4.8	8.1	(3.4, 16)	**1.1E-05**
Active Enhancer Flank (EnhAF)	3	1.8	3.6	(0.72, 11)	0.055
Weak Enhancer 1 (EnhW1)	12	7.3	3.7	(1.9, 6.7)	**1.9E-04**
Weak Enhancer 2 (EnhW2)	16	9.7	3.3	(1.8, 5.5)	**8.0E-05**
Enhancer Acetylation Only (EnhAc)	0	0.0	0.0	(0, 44)	1
DNase only (DNase)	1	0.61	0.96	(0.024, 5.4)	1
ZNF genes & repeats (ZNF/Rpts)	0	0.0	0.0	(0, 13)	1
Heterochromatin (Het)	0	0.0	0.0	(0, 3.6)	0.63
Poised Promoter (PromP)	2	1.2	1.1	(0.13, 3.9)	0.71
Bivalent Promoter (PromBiv)	11	6.7	0.85	(0.41, 1.6)	0.77
Repressed Polycomb (ReprPC)	9	5.5	0.75	(0.34, 1.5)	0.54
Quiescent/Low (Quies)	15	9.1	0.22	(0.12, 0.37)	6.7E-12

Supplementary Table 19. Enrichment of the 25 chromatin states among 2,093 candidate CpGs for GA-involved epigenetic memory in the analysis using cord blood T cell-based and B cell-based annotation provided by ChromHMM.

*Bold characters mean enriched chromatin states which met the criteria of Bonferroni-significance (*p*-value <0.05/25) and odds ratio ≥ 1. **OR*: odds ratio, *CI*: confidence interval

Following Abbreviations of 25 chromatin states are defined in ChromHMM; TssA: Active TSS, PromU: Promoter upstream TSS, PromD1: Promoter downstream with DNase, PromD2: Promoter downstream TSS, Tx5': Transcription 5', Tx: Transcription, Tx3': Transcription 3', TxWk: Weak Transcription, TxReg: Transcription Regulatory, TxEnh5': Transcription 5' Enhancer, TxEnh3': Transcription 3' Enhancer, TxEnhW: Transcription Weak Enhancer, EnhA1: Active Enhancer 1, EnhA2: Active Enhancer 2, EnhAF: Active Enhancer Flank, EnhW1: Weak Enhancer 1, EnhW2: Weak Enhancer 2, EnhAc: Enhancer Acetylation Only, DNase: DNase only, ZNF/Rpts: ZNF genes & repeats, Het: Heterochromatin, PromP: Poised Promoter, PromBiv: Bivalent Promoter, ReprPC: Repressed Polycomb, Qies: Quiescent/Low.

[T cell-based annotation]

25-Chromatin-State	Proportion (%)	OR	95%CI	*p*-value
Active TSS (TssA)	0.2	0.034	(0.0094, 0.088)	1.3E-42
Promoter upstream TSS (PromU)	8.9	0.82	(0.70, 0.96)	0.010
Promoter downstream with DNase (PromD1)	3.6	0.31	(0.25, 0.40)	4.8E-32
Promoter downstream TSS (PromD2)	2.4	2.5	(1.9, 3.3)	**1.4E-08**
Transcription 5' (Tx5')	1.0	0.51	(0.31, 0.79)	0.0014
Transcription (Tx)	0.2	0.12	(0.037, 0.27)	7.9E-13
Transcription 3' (Tx3')	1.0	0.18	(0.11, 0.29)	2.7E-24
Weak Transcription (TxWk)	2.1	0.57	(0.41, 0.77)	8.1E-05
Transcription Regulatory (TxReg)	1.2	1.1	(0.70, 1.6)	0.68
Transcription 5' Enhancer (TxEnh5')	0.3	0.53	(0.20, 1.2)	0.13
Transcription 3' Enhancer (TxEnh3')	0.9	1.3	(0.79, 2.1)	0.23
Transcription Weak Enhancer (TxEnhW)	1.0	0.51	(0.31, 0.80)	0.0014

Active Enhancer 1 (EnhA1)	0.9	2.2	(1.3, 3.6)	0.0020
Active Enhancer 2 (EnhA2)	0.9	1.4	(0.81, 2.2)	0.17
Active Enhancer Flank (EnhAF)	0.4	0.83	(0.38, 1.6)	0.76
Weak Enhancer 1 (EnhW1)	5.6	2.8	(2.3, 3.4)	**5.9E-21**
Weak Enhancer 2 (EnhW2)	4.2	1.3	(1.1, 1.6)	0.012
Enhancer Acetylation Only (EnhAc)	0	0	(0, 3.5)	0.63
DNase only (DNase)	1.5	2.4	(1.6, 3.4)	**2.4E-05**
ZNF genes & repeats (ZNF/Rpts)	0.2	1.1	(0.29, 2.7)	0.79
Heterochromatin (Het)	0.5	0.76	(0.37, 1.4)	0.49
Poised Promoter (PromP)	3.4	3.1	(2.4, 3.9)	**1.4E-15**
Bivalent Promoter (PromBiv)	14.0	1.9	(1.7, 2.2)	**3.0E-22**
Repressed Polycomb (ReprPC)	19.3	3.1	(2.8, 3.5)	**3.5E-73**
Quiescent/Low (Quies)	26.5	0.79	(0.71, 0.86)	3.1E-07

[B cell-based annotation]

25-Chromatin-State	Proportion (%)	OR	95%CI	*p*-value
Active TSS (TssA)	0.6	0.11	(0.056, 0.18)	3.1E-35
Promoter upstream TSS (PromU)	9.7	0.95	(0.82, 1.1)	0.51
Promoter downstream with DNase (PromD1)	6.6	0.48	(0.40, 0.57)	4.2E-20
Promoter downstream TSS (PromD2)	2.1	3.2	(2.3, 4.4)	**6.6E-11**
Transcription 5’ (Tx5’)	0.6	0.30	(0.15, 0.52)	4.6E-07
Transcription (Tx)	0.5	0.21	(0.10, 0.39)	2.0E-10
Transcription 3’ (Tx3’)	1.3	0.21	(0.14, 0.30)	6.4E-27
Weak Transcription (TxWk)	2.0	0.55	(0.40, 0.75)	3.4E-05
Transcription Regulatory (TxReg)	1.6	1.1	(0.78, 1.6)	0.46
Transcription 5’ Enhancer (TxEnh5’)	0.4	0.52	(0.24, 0.99)	0.051
Transcription 3’ Enhancer (TxEnh3’)	0.8	1.2	(0.70, 1.9)	0.42
Transcription Weak Enhancer (TxEnhW)	0.7	0.53	(0.29, 0.89)	0.013
Active Enhancer 1 (EnhA1)	0.4	0.68	(0.29, 1.4)	0.37
Active Enhancer 2 (EnhA2)	0.6	0.74	(0.38, 1.3)	0.38

Active Enhancer Flank (EnhAF)	0.4	0.71	(0.33, 1.4)	0.39
Weak Enhancer 1 (EnhW1)	3.9	2.8	(2.2, 3.5)	**6.8E-15**
Weak Enhancer 2 (EnhW2)	2.9	1.2	(0.92, 1.6)	0.15
Enhancer Acetylation Only (EnhAc)	0.5	1.3	(0.64, 2.3)	0.39
DNase only (DNase)	1.8	2.7	(1.9, 3.8)	**8.7E-08**
ZNF genes & repeats (ZNF/Rpts)	0.2	0.82	(0.22, 2.1)	1
Heterochromatin (Het)	0.7	0.63	(0.34, 1.1)	0.086
Poised Promoter (PromP)	2.3	2.4	(1.7, 3.1)	**1.3E-07**
Bivalent Promoter (PromBiv)	14.5	2.3	(2.0, 2.6)	**1.3E-33**
Repressed Polycomb (ReprPC)	20.8	3.0	(2.7, 3.3)	**1.3E-72**
Quiescent/Low (Quies)	24.2	0.79	(0.71, 0.87)	1.7E-06

Supplementary Table 20. Transcription-correlated & GA-involved epigenetic memory candidate CpGs.

CpGprobe	Chr.State (CB, T cell)	Gene	Corr.Exp	Chr	MAPINFO	feature	%Meth/w (%/week)	pval.GA	Corr. CB-PB
			N = 55				N = 110	N =110	N = 47
cg26411822	2_PromU	*STX12*	-0.29	1	28099013	TSS1500	-0.7	4.3E-05	0.70
cg00320094	24_ReprPC	*GPR177*	0.41	1	68695183	Body	0.7	8.4E-08	0.72
cg02987481	24_ReprPC	*NGF*	-0.41	1	115881544	TSS1500	0.3	5.9E-05	0.73
cg07790079	25_Quies	*(NGF)*	-0.32	1	116021925	IGR	0.4	6.5E-05	0.82
cg25599129	25_Quies	*(NGF)*	-0.32	1	116022006	IGR	0.7	1.0E-05	0.82
cg16246545	4_PromD2	*PHGDH*	-0.52	1	120255941	Body	0.5	1.8E-07	0.70
cg27179622	25_Quies	*LEFTY2*	-0.30	1	226127290	Body	0.9	1.3E-04	0.94
cg20028470	24_ReprPC	*UCN*	0.44	2	27530829	5'UTR	0.3	3.8E-06	0.87
cg07537370	24_ReprPC	*UCN*	0.39	2	27531124	5'UTR	0.4	4.8E-04	0.79
cg05231308	24_ReprPC	*UCN*	0.41	2	27531163	TSS200	0.6	2.1E-09	0.84
cg04527918	24_ReprPC	*UCN*	0.53	2	27531170	TSS200	0.6	4.7E-07	0.82
cg13833437	24_ReprPC	*UCN*	0.46	2	27531236	TSS200	0.6	1.1E-06	0.81
cg05113927	24_ReprPC	*UCN*	0.39	2	27531244	TSS200	0.6	3.7E-06	0.81
cg21752601	24_ReprPC	*UCN*	0.43	2	27531310	TSS200	0.5	4.7E-07	0.72
cg20442078	24_ReprPC	*UCN*	0.46	2	27531360	TSS1500	1.1	4.6E-12	0.73
cg05960677	2_PromU	*RBM43*	-0.33	2	152117363	Body	-1.8	5.0E-09	0.70
cg26627956	6_Tx	*CFLAR*	0.31	2	202004766	Body	0.7	3.7E-08	0.75
cg21674813	25_Quies	*OBSL1*	-0.35	2	220425073	Body	0.7	3.9E-08	0.76
cg01948202	3_PromD1	*PARP14*	-0.32	3	122400474	Body	-1.3	1.3E-07	0.82
cg00509649	9_TxReg	*SLC12A7*	-0.61	5	1103848	Body	-1.3	1.6E-03	0.92
cg13681701	9_TxReg	*SLC12A7*	-0.58	5	1103910	Body	-0.8	5.8E-04	0.90
cg16163535	13_EnhA1	*SLC12A7*	-0.59	5	1107098	Body	-0.8	1.6E-04	0.71
cg14596589	13_EnhA1	*SLC12A7*	-0.44	5	1107148	Body	-0.9	4.1E-04	0.83
cg13592947	3_PromD1	*SLC12A7*	-0.45	5	1111049	Body	-0.6	2.6E-03	0.93
cg11033617	25_Quies	*RASGEF1C*	-0.28	5	179562118	Body	-0.2	1.0E-03	0.81
cg17099072	2_PromU	*GABBR1*	0.40	6	29601489	TSS1500	0.6	2.0E-05	0.71
cg18221076	17_EnhW2	*(HCG26)*	0.52	6	31442934	IGR	0.5	9.0E-05	0.83
cg17362900	17_EnhW2	*HLA-DPB1*	0.27	6	33047944	Body	0.9	5.1E-08	0.71
cg01418527	17_EnhW2	*FSCN1*	-0.47	7	5631189	TSS1500	0.3	7.8E-04	0.74
cg02557364	25_Quies	*(TAS2R41)*	-0.30	7	143208268	IGR	0.2	1.4E-05	0.76
cg08384657	25_Quies	*(TAS2R41)*	-0.28	7	143208406	IGR	0.6	1.0E-08	0.73

cg20490392	16_EnhW1	*ANGPT2*	-0.33	8	6419438	Body	1.2	2.8E-04	0.76
cg25350011	16_EnhW1	*ANGPT2*	-0.33	8	6419483	Body	1.2	4.9E-04	0.83
cg04017131	16_EnhW1	*ANGPT2*	-0.32	8	6419570	Body	1.0	1.2E-03	0.87
cg04259752	23_PromBiv	*LOXL2*	0.47	8	23262159	TSS1500	0.6	2.3E-10	0.76
cg02174341	25_Quies	*UCMA*	0.38	10	13276466	TSS200	0.7	2.3E-09	0.71
cg02737384	2_PromU	*RASSF4*	-0.39	10	45470199	Body	0.6	3.6E-03	0.87
cg12527260	23_PromBiv	*RCOR2*	-0.40	11	63683744	Body	0.3	8.5E-05	0.80
cg17205324	24_ReprPC	*EFS*	-0.47	14	23835595	TSS1500	0.4	5.7E-12	0.80
cg19628497	24_ReprPC	*DLK1*	-0.81	14	101194267	Body	0.8	1.7E-03	0.93
cg22502625	24_ReprPC	*C14orf73*	-0.31	14	103568366	Body	0.3	1.8E-03	0.78
cg10973146	24_ReprPC	*C14orf73*	-0.35	14	103568471	Body	0.7	7.9E-07	0.72
cg09814127	5_Tx5'	*TNFAIP2*	0.35	14	103593235	Body	0.5	3.1E-04	0.89
cg18587137	5_Tx5'	*TNFAIP2*	0.30	14	103593503	Body	0.9	1.9E-05	0.87
cg18620571	5_Tx5'	*TNFAIP2*	0.34	14	103593505	Body	0.7	1.8E-04	0.91
cg10501093	5_Tx5'	*TNFAIP2*	0.33	14	103593520	Body	0.8	1.8E-04	0.89
cg05899103	8_TxWk	*LOC390595*	0.58	15	65394570	Body	0.7	9.7E-09	0.72
cg24339470	16_EnhW1	*SARM1*	0.34	17	26712412	Body	0.2	3.3E-05	0.72
cg08577293	19_DNase	*DHX58*	0.54	17	40253614	3'UTR	0.7	1.9E-07	0.72
cg00441918	19_DNase	*IGF2BP1*	-0.49	17	47113407	Body	0.5	7.1E-05	0.78
cg00376735	8_TxWk	*LPPR2*	0.35	19	11473212	Body	1.1	5.0E-10	0.73
cg19699893	8_TxWk	*LPPR2*	0.29	19	11473353	Body	0.4	7.2E-08	0.71
cg17250863	8_TxWk	*GGT7*	0.58	20	33451272	Body	1.0	8.3E-12	0.75
cg23835646	2_PromU	*LGALS2*	-0.49	22	37976063	TSS200	-0.4	4.6E-05	0.73

*1) Chr.State (CB, T cell): the 25 chromatin states which were imputed on the CpG of cord blood T cell by ChromHMM.

*2) Gene: gene which has the CpG on it. Gene names in parenthesis mean the genes whose transcriptions were considered to be associated with the neighborhood intergenic CpG methylation.

*3) Corr.Exp: correlation coefficient between %methylation and log2-transformed expression of corresponding gene. The values were generated in methylation-expression-analysis using 55 cord blood samples.

*4) Chr: chromosome number

*5) MAPINFO: genomic coordinate.

*6) feature: genomic feature annotated by Illumina. Here, “Promoter Region” includes TSS200, TSS1500, 5’UTR, 1stExon, while “Gene Body Region” includes Body, 3’UTR. IGR means intergenic region.

*7) %Meth/w (%/week): %methylation change per a gestational week advance. The values were generated as regression coefficients in cord blood EWAS using 110 samples.
*8) pval.GA: *p*-value generated in cord blood EWAS on GA using 110 samples.
*9) Corr. CB-PB: correlation coefficient between cord blood and postnatal blood methylation. The values were generated in the analysis using the methylation data of 47 babies who provided both cord blood and postnatal blood samples.
***GA*: gestational age
Following Abbreviations are defined in ChromHMM; 1_TssA: Active TSS, 2_PromU: Promoter upstream TSS, 3_PromD1: Promoter downstream with DNase, 4_PromD2: Promoter downstream TSS, 5_Tx5': Transcription 5', 6_Tx: Transcription, 7_Tx3': Transcription 3', 8_TxWk: Weak Transcription, 9_TxReg: Transcription Regulatory, 10_TxEnh5': Transcription 5' Enhancer, 11_TxEnh3': Transcription 3' Enhancer, 12_TxEnhW: Transcription Weak Enhancer, 13_EnhA1: Active Enhancer 1, 14_EnhA2: Active Enhancer 2, 15_EnhAF: Active Enhancer Flank, 16_EnhW1: Weak Enhancer 1, 17_EnhW2: Weak Enhancer 2, 18_EnhAc: Enhancer Acetylation Only, 19_DNase: DNase only, 20_ZNF/Rpts: ZNF genes & repeats, 21_Het: Heterochromatin, 22_PromP: Poised Promoter, 23_PromBiv: Bivalent Promoter, 24_ReprPC: Repressed Polycomb, 25_Qies: Quiescent/Low.

[References]

1 Heinz, S. *et al.* Simple combinations of lineage-determining transcription factors prime cis-regulatory elements required for macrophage and B cell identities. *Molecular cell* **38**, 576-589, doi:10.1016/j.molcel.2010.05.004 (2010).

2 Benjamini, Y. & Hochberg, Y. Controlling the false discovery rate: a practical and powerful approach to multiple testing. *Journal of the royal statistical society. Series B (Methodological)*, 289-300 (1995).

3 Huang da, W., Sherman, B. T. & Lempicki, R. A. Systematic and integrative analysis of large gene lists using DAVID bioinformatics resources. *Nature protocols* **4**, 44-57, doi:10.1038/nprot.2008.211 (2009).

4 Agha, G. *et al.* Birth weight-for-gestational age is associated with DNA methylation at birth and in childhood. *Clinical epigenetics* **8**, 118, doi:10.1186/s13148-016-0285-3 (2016).

5 Ernst, J. & Kellis, M. Large-scale imputation of epigenomic datasets for systematic annotation of diverse human tissues. *Nature biotechnology* **33**, 364-376, doi:10.1038/nbt.3157 (2015).

6 Schroeder, J. W. *et al.* Neonatal DNA methylation patterns associate with gestational age. *Epigenetics* **6**, 1498-1504, doi:10.4161/epi.6.12.18296 (2011).

7 Simpkin, A. J. *et al.* Longitudinal analysis of DNA methylation associated with birth weight and gestational age. *Human molecular genetics* **24**, 3752-3763, doi:10.1093/hmg/ddv119 (2015).

8 Bohlin, J. *et al.* Prediction of gestational age based on genome-wide differentially methylated regions. *Genome biology* **17**, 207, doi:10.1186/s13059-016-1063-4 (2016).

9 Knight, A. K. *et al.* An epigenetic clock for gestational age at birth based on blood methylation data. *Genome biology* **17**, 206 (2016).
